# The impact of treatment strategies on the epidemiological dynamics of plasmid-conferred antibiotic resistance

**DOI:** 10.1073/pnas.2406818121

**Published:** 2024-12-17

**Authors:** Malte Muetter, Daniel C. Angst, Roland R. Regoes, Sebastian Bonhoeffer

**Affiliations:** ^a^Institute of Integrative Biology, Department for Environmental System Science, ETH Zurich, Zurich 8092, Switzerland

**Keywords:** combination therapy, antibiotic resistance, plasmids, experimental epidemiology, treatment strategies

## Abstract

The issue of antibiotic resistance is a critical concern for public health, prompting numerous investigations into the impact of treatment strategies on preventing or slowing down the emergence of resistance. While existing studies have predominantly focused on chromosomal resistance mutations, the consequences of often clinically more relevant plasmid-conferred resistance remain insufficiently explored. To address this gap, we conducted three extensive in vitro experiments utilizing a liquid-handling platform. These experiments evaluated the efficacy of five distinct treatment strategies using two antibiotics (tetracycline and ceftazidime) along with two horizontally transmissible clinical resistance plasmids conferring the respective resistances. Among the experimentally investigated treatment strategies, combination therapy proved to be the most effective in preventing the emergence of double resistance while minimizing the number of infections. To verify the reliability of these findings, we constructed a computational model of our experiments that we parameterized using the experimental data. We employed this model to augment the experimental data by conducting an in silico parameter sensitivity analysis. The sensitivity analysis corroborated our experimental results, demonstrating that combination therapy consistently outperformed other treatment strategies across a range of parameter values.

## Significance

Given the rising levels of antibiotic resistance, we need a better understanding of how to use antibiotics sustainably. While most current work on treatment strategies to reduce resistance focuses on chromosomal resistance, we focus on plasmid-borne resistance, which can be transmitted between bacteria. We combine large-scale in vitro experiments with a computational model to assess the effectiveness of various treatment strategies in managing preexisting antibiotic resistance and preventing the emergence of new double resistance through horizontal gene transfer. We observed that combination therapy is most effective in suppressing the emergence of plasmid-borne double resistance. This is achieved by decreasing cocolonization with single-resistant strains and reducing the probability of cocolonized patients developing double resistance.

In light of the growing threat of antimicrobial resistance (AMR) to human health, various multidrug strategies are being considered to improve the sustainability of antibiotic use. These approaches include combination therapy (simultaneous use of multiple antibiotics), mixing therapy (randomly assigning patients to receive different antibiotics), and cycling therapy (alternating between multiple antibiotics over time).

Combination, originally proposed alongside cycling therapy to prevent biocide resistance in plant pathogens (1–3), was later adopted in human medicine. Combination therapy proved its effectiveness in preventing resistance evolution in highly adaptable pathogens such as HIV, *Mycobacterium tuberculosis*, and *Plasmodium falciparum* (4). However, a recent meta-analysis investigating the effect of combination therapy on resistance across various bacterial infections and antibiotic combinations found no evidence for a difference in the risk of resistance acquisition (5). Also, a comprehensive cluster-randomized crossover study comparing mixing and cycling by van Duijn et al. (6), spanning nearly 2 y across eight ICUs, found no significant differences in outcomes.

A review of the available model literature by Uecker et al. (7) reveals the complexity and context-dependent efficacy of treatment strategies such as combination, cycling, or mixing strategies. Yet, theoretical models often identify combination therapy as the best strategy to prevent new resistance (8, 9). It remains unclear whether the inconclusive results regarding the effectiveness of multidrug treatment strategies in the literature are due to the theoretical models failing to account for key processes, or if clinical studies lack statistical power, as suggested by Siedentop et al. (5). This lack of power may be caused by patient and bacterial strain heterogeneity, stochasticity in infection dynamics, and other unknown factors that make it difficult to isolate single effects.

We recently started experiments to make a foray into the large gap between theoretical models and clinical trials. In an in vitro experiment mimicking the epidemiological scenario of transmission in a hospital ward, Angst et al. (10) investigated the effect of cycling, mixing, and combination therapy on resistance evolution and showed that for chromosomal resistance mutations combination therapy outperformed the other strategies. One potential reason why combination therapy succeeded in that study and tends to be superior in mathematical models is that it increases the genetic barrier to resistance by requiring the acquisition of multiple mutations in the same background.

Here, we explore the effect of horizontal gene transfer (HGT) on resistance evolution under treatment by conducting three large-scale in vitro experiments. The experiments mimic epidemiological transmission dynamics of symptomatic infections by a focal strain in an intensive care unit (ICU) and include patient discharge and admission, infection between patients, and treatment. We use two antibiotics, ceftazidime (A) and tetracycline (B), along with two clinical resistance plasmids (11), we call $p_{A}$ and $p_{B}$, conferring resistance to the corresponding antibiotics. The plasmids are compatible, can conjugate, and were isolated from clinical samples collected and characterized in a study at University Hospital Basel (12). We model patients as wells in a 384-well microtiter plate filled with LB medium. These “patients” can be infected with a mixture of bacteria, which may carry up to two resistance plasmids. Depending on the presence of bacteria and resistance, we assign each patient a resistance profile: uninfected (U), sensitive infected (S), single-resistant ($A_{r}$, $B_{r}$, or $\left(A_{r}\&B_{r}\right)$), or double-resistant ($AB_{r}$).

In each experiment, we model six hospital wards to assess the ability of five treatment strategies (mixing, cycling, combination therapy with two antibiotics and two monotherapies with each antibiotic alone) and one control (no antibiotics) to contain the spread of plasmid-borne resistance and prevent the emergence of double resistance. All patients in each ward are treated daily according to the assigned strategy. A schematic of the experimental setup is shown in *SI Appendix*, Fig. S1. Each of the three experiments addresses a different scenario (Table 1), varying in patient turnover probability (admission/discharge), infection probability, and the distribution of resistance profiles for incoming patients (sampling proportions). The prevention scenario addresses a situation with low levels of preexisting single and no double resistance brought into the hospital ward from the community. The containment scenario focuses on the ability of treatment strategies to contain preexisting double resistance and in the maximum-emergence scenario, we maximized the opportunities for emerging double resistance through HGT by admitting single-resistant patients only.

**Table 1.** Parameter sets and $R_{0}$ used in the three experiments: $c_{\phi }$ is the proportion of admitted patients with resistance profile ϕ, τ denotes the probability that a patient is replaced with a new patient sampled from the community and β denotes the infection probability

Scenario	$c_{S}$	$c_{\mathrm{Ar}}$	$c_{\mathrm{Br}}$	$c_{\mathrm{ABr}}$	$c_{U}$	τ	β	$R_{0}$
Prevention	0.75	0.05	0.05	0	0.15	0.20	0.30	1.5
Containment	0.58	0.11	0.11	0.05	0.15	0.20	0.35	1.75
Maximum-emergence	0	0.50	0.50	0	0	0.50	0.25	0.5

Alongside our experiments, we created a computational model that mimics the experiment and is parameterized but not fitted using the experimental data. We used the model to assess the robustness of our findings to the randomization of the experimental decisions and conducted an in silico sensitivity analysis to augment the experimental data.

## Results

In each of our three experiments, we simulated the transition dynamics across six hospital wards on six 384-well plates. Each 384-well plate simulates four replicate hospital wards, with each replicate comprising 96 wells representing 94 patients and two negative controls. We replace each assay plate daily to renew the treatment and medium (*SI Appendix*, Fig. S1). Based on the turnover probability τ, we randomly decide whether a patient stays. If this is the case, we inoculate the well on the new plate from the same well on the old plate. Else we replace this patient with a new incoming patient by inoculating the well on the new plate from a strain plate containing all resistance profiles. The resistance profile of the incoming patient is randomly selected based on predefined probabilities (sampling proportions $c_{\phi }$). Based on the infection probability β, we randomly decide whether a patient will infect another randomly chosen patient. These infections are then simulated in vitro by passing a drop to the infected well on the new plate. All inoculations are carried out using the same pintool.

### Multidrug Strategies Keep the Overall Number of Infections Lowest and Best Suppress Single Resistance.

**Fig. 1.**
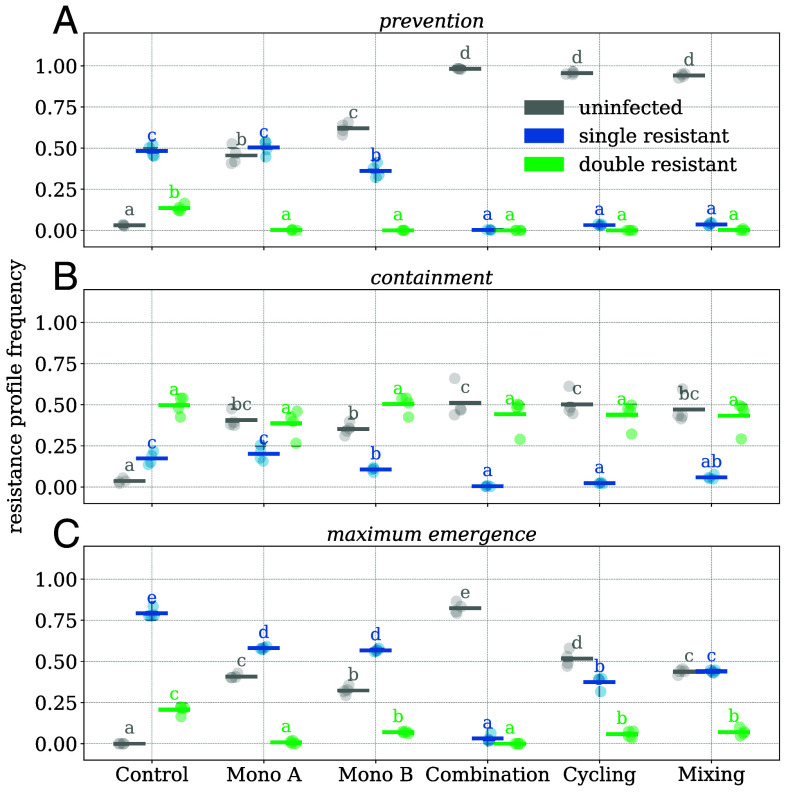
Panels (*A*–*C*) show the mean frequency of uninfected (gray), single-resistant infected (blue), and double-resistant infected wells (green) during the last four transfers of the three scenarios. Circles represent replicates (n = 4), and bars represent means. Within resistance categories, bars not sharing a letter are significantly different (pairwise Tukey post hoc test, P<0.05; ANOVA tables and all *P*-values can be found in *SI Appendix*, Tables S34–S50).

The prevention scenario is characterized by a moderate proportion of single-resistant admissions to the hospital ward, the absence of preexisting double resistance, and a moderately spreading infection dynamic ($R_{0}=1.5$, *SI Appendix*, Eq. **S1** and *SI Methods*). In this scenario, there are no differences between combination, mixing, and cycling on the frequency of uninfected, single-resistant-infected, and double-resistant-infected wells (Fig. 1*A*, time series in *SI Appendix*, Fig. S2).

However, all multidrug strategies are significantly better at suppressing single resistance and increasing the number of cleared wells than the single-drug strategies and the control without treatment (Fig. 1*A*). In all scenarios, combination therapy was one of the most successful treatment strategies in minimizing single-resistant and overall infections. At the same time, we observed most single and double resistance in the untreated control. All strategies (but not the control) were able to clear sensitive infections effectively with clearance probabilities of 97% for drug A, 73% for drug B, and 86% for AB (*SI Appendix*, Table S8).

### All Treatment Strategies Fail to Contain Preexisting Double Resistance.

The containment scenario explores a situation in which patients infected with double-resistant bacteria are continuously admitted to the hospital. No strategy was able to contain the spread of double resistance, resulting in increased frequencies of double resistance (>40%) in all treatment arms at the conclusion of the experiment (Fig. 1*B*).

### Treatment Strategies Affect the Emergence of Double Resistance.

In our experiments, double resistance primarily emerges in wells inoculated with both single-resistance plasmids via HGT, as the evolution of de novo resistance (e.g., by point mutations) to high drug concentrations (>50×MIC) is unlikely. As the inoculum volumes for turnover, infection, and passage are identical in our experiments, we do not distinguish between wells containing A-resistance ($A_{r}$) infecting wells containing B-resistance ($B_{r}$) or vice versa and simply refer to these events as superinfections.

**Fig. 2.**
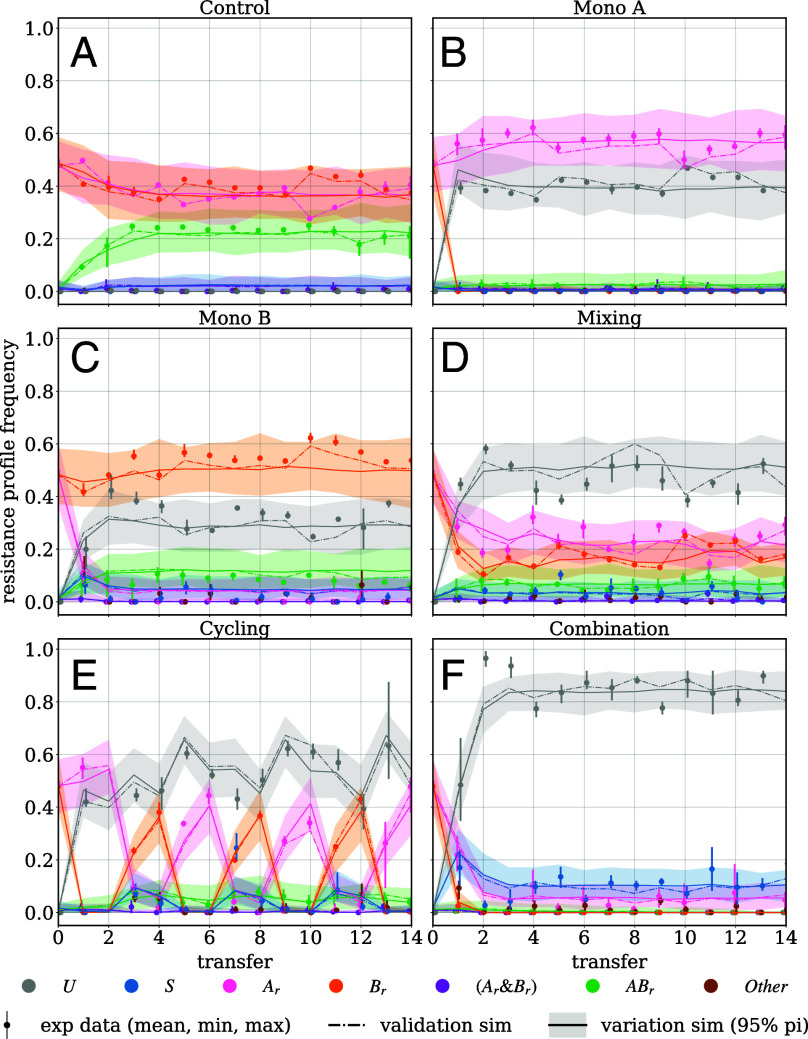
Frequencies of resistance profiles over time during the maximum-emergence scenario. Panels (*A*–*F*) show the six tested strategies. Dots and bars show the mean and min/max interval of the four replicates. The dash-dotted line shows the mean value of 100 stochastic simulations based on the instruction set used in the in vitro experiment (Validation simulation). The solid line shows the mean value of 100 simulations with randomly created instruction sets based on the parameter set used in the experiment (Variation simulations). The shaded error band indicates the 95-percentile interval between the Variation simulations.

During the prevention and containment scenario, we could not identify differences in the strategies’ abilities to suppress the emergence of double resistance. We attribute this to a lack of statistical power because we observed only a few instances of double resistance emerging, mostly in the untreated control. To address this, we selected parameters for the maximum-emergence scenario designed to maximize superinfection opportunities between wells carrying complementary resistance. To this end, all admitted patients carried bacteria with only one of the two resistance plasmids (at equal proportions). In addition, we set the probability of admission and discharge to τ=0.5 and the infection probability to β=0.25, resulting in a basic reproduction number $R_{0}=0.5$ (*SI Appendix*, Eq. **S1**). An $R_{0}<1$ makes double resistance more likely to be replaced by newly admitted patients than to spread, thus maintaining a high potential for emergence. We implement this scenario to explore emergence under a magnifying glass, being aware that it does not reflect a likely clinical situation. In this scenario, combination therapy and monotherapy with drug A lead to the lowest frequency of double resistance during the last four transfers (Figs. 1*C* and 2).

**Fig. 3.**
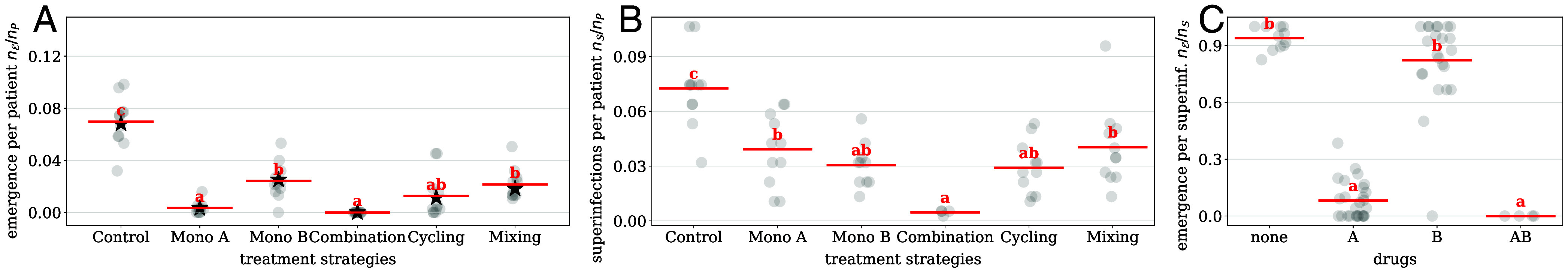
Analysis of the emergence of double resistance in vitro and superinfection between single resistant $A_{r}$ and $B_{r}$ wells during the maximum-emergence scenario, from transfer four onward. Each dot corresponds to data from a single plate, with each plate representing a distinct treatment arm, encompassing 376 patients for one transfer. Mean values are represented by red bars. Bars not sharing a letter are significantly different (P<0.05, ANOVA tables and pairwise Tukey post hoc results can be found in *SI Appendix*, Tables S51–S56). (*A*) number of newly emerged cases of double resistance per plate ($n_{E}$), normalized to the total number of patients (wells) per plate ($n_{P}=376$). Here, the stars indicate predictions based on the ratio of superinfections per patient and the emergence per superinfection (*SI Appendix*, *SI Computational Model*). (*B*) Number of superinfections per plate ($n_{S}$), normalized to the total number of patients (wells) per plate ($n_{P}=376$). (*C*) Number of superinfected wells treated with (A, B, AB, and none) that develop double resistance ($n_{E}$) divided by the number of all superinfected wells ($n_{S}$) receiving the respective treatment.

For the maximum-emergence scenario, we observed that combination therapy, cycling, and monotherapy with drug A were most effective in preventing newly emerging double resistance. Combination was the only strategy in which we did not observe a single case of emerging double resistance after the first transfer (Fig. 3*A*). Furthermore, combination therapy is the most successful treatment strategy in minimizing the number of both single-resistant and overall infections, while the control leads to the highest number of double- and single-resistant, and overall infections.

### Combination Therapy Suppresses the Emergence of Double Resistance by Preventing Superinfections.

Treatment strategies can impact the emergence of double resistance by suppressing superinfections. The number of superinfections $n_{S}$ is dependent on the abundance of the single resistance carrying wells $A_{r}$ and $B_{r}$. Hence, we expect the highest number of superinfections and most opportunities for emerging double resistance when both single resistances are unaffected by the treatment and the fewest if the treatment successfully suppresses both single resistances. Our measurements confirmed these expectations during the maximum-emergence scenario. Here, $n_{S}$ is highest in the control (no treatment) and lowest under combination therapy (Fig. 3*B*).

### Treatment Strategies Influence the Emergence of Double Resistance within Superinfected Wells.

We observed the highest average frequency of superinfections developing double resistance $\left(\frac{n_{E}}{n_{S}}\right)$ in antibiotic-free medium and in medium treated with antibiotic B (tetracycline). In contrast, superinfections resulting in double resistance rarely occur in medium treated with antibiotic A (ceftazidime) or both drugs (Fig. 3*C*). We think the impact of treatment on cell densities within superinfected wells (both in infected and infecting wells) can best explain these findings.

First, applying a drug affects the in-well population dynamics of superinfected wells. Reducing the cell density for one or both single-resistant strains within a superinfected well reduces the probability of bacteria with complementary resistance to encounter and conjugate (*SI Appendix*, *SI Results*). As drug A (bactericidal) decreases the cell density faster than drug B (bacteriostatic), more conjugation opportunities occur in wells treated with drug B.

Second, the treatment strategies influence the number of transferred single-resistant bacteria that inoculate superinfections by curbing the bacterial density within the infecting wells (*SI Appendix*, *SI Results*).

Due to the differences in the abilities of drugs A and B to prevent conjugation, there are times (cycles) and places (beds) during cycling and mixing where using drug B offers increased opportunities for the emergence of double resistance, which is never the case with combination therapy.

### Computational Model Corroborates the Robustness of Experimental Outcomes.

The experiments are conducted by a liquid handling platform that carries out predefined instructions, specifying which infections occur and who is admitted or discharged. The instructions are randomized based on parameter sets we defined for each scenario, including the overall infection and turnover probability as well as the distribution of the resistance profiles of admitted patients. We call the entirety of all instructions that come up during one experiment an “instruction set.” Due to the scale and technical complexity of the experiments, it was not feasible to carry out individual instruction sets for each replicate, so we opted to apply the same instruction sets for all replicates. This raises the question of whether the experimental results are a consequence of a specific instance of this random process and whether they are robust to the randomization in the instruction set. To address this, we developed a discrete-time stochastic model comprising 94 individual in silico patients mimicking the epidemiological dynamics of the experiment (*SI Appendix*, *SI Computational Model*). The model was parametrized, but not fitted, with transition probabilities (*SI Appendix*, Tables S18–S25) that we estimated based on the transition frequencies measured in vitro. We used the same transition probabilities in the simulations for all scenarios.

First, we validated the model by averaging 100 validation simulations, each employing the identical instruction sets used in vitro. The aim of the validation simulations is to recreate the experiments in silico (*SI Appendix*, Fig. S3*B*). We found that the simulation results are in good agreement with the experimental data, indicating that the model reflects the dynamics observed in the in vitro experiments well (Fig. 2 and *SI Appendix*, Figs. S2 and S4). One exception is the spread of A-resistance during the prevention scenario in control and Mono A. This could indicate an increased number of contaminations at the beginning of the prevention scenario. We also observe some discrepancies for the spread of double resistance during the prevention scenario, which we attribute to contamination artifacts in the transition probabilities (*SI Appendix*, *SI Computational Model*).

Second, we averaged 100 variation simulations to assess the robustness of the experimental outcomes against variations in the instruction sets. In these variation simulations, each of the 100 instruction sets was randomized based on the same three parameter sets used in vitro (*SI Appendix*, Fig. S3*C*). Differences between the validation and variation simulations indicate differences in outcome due to the randomization of the instruction sets. For instance, with a turnover probability τ=0.2 and an admission probability $c_{A}=0.05$, we expect 0.94 $A_{r}$ admissions per transfer. However, random fluctuations can result in either more (or fewer) $A_{r}$ admissions, leading to a temporarily higher (or lower) frequency of $A_{r}$ in the validation simulations, creating a temporary spread between the validation and variation simulations. We observed that the validation simulations fluctuate around the variation simulations and never diverge far (Fig. 2 and *SI Appendix*, Figs. S2 and S4), indicating robustness of the experimental results to the randomization of the instruction sets.

### In Silico Sensitivity Analysis Indicates that the Superiority of Combination Therapy Is Robust.

Given that the validation simulations agreed well with the experiments, we used the model to perform an in silico parameter sensitivity analysis of the experimental results (*SI Appendix*, Fig. S3*D*). To this end, we ran ten simulations for each of 20,000 randomly generated parameter sets by varying the turnover and infection probability and the five sampling proportions for incoming patients: τ, β, $c_{S}$, $c_{A_{r}}$, $c_{B_{r}}$, $c_{AB_{r}}$, and $c_{U}$. For half of the parameter sets, we forced the frequency of incoming patients with double resistance ($c_{AB_{r}}$) to zero.

We used the frequency of uninfected in silico patients to measure treatment success. Using this criterion, the control strategy (no treatment) always performed worst, and accordingly, we excluded this treatment arm from the following analysis. Strategies were then classified as i) “single winners” if they are significantly better than all other strategies; ii) “winners” if they are not outperformed by any other strategy; iii) “losers” if they do not outperform at least one other strategy; or iv) “single losers” if all other strategies outperform them.

**Fig. 4.**
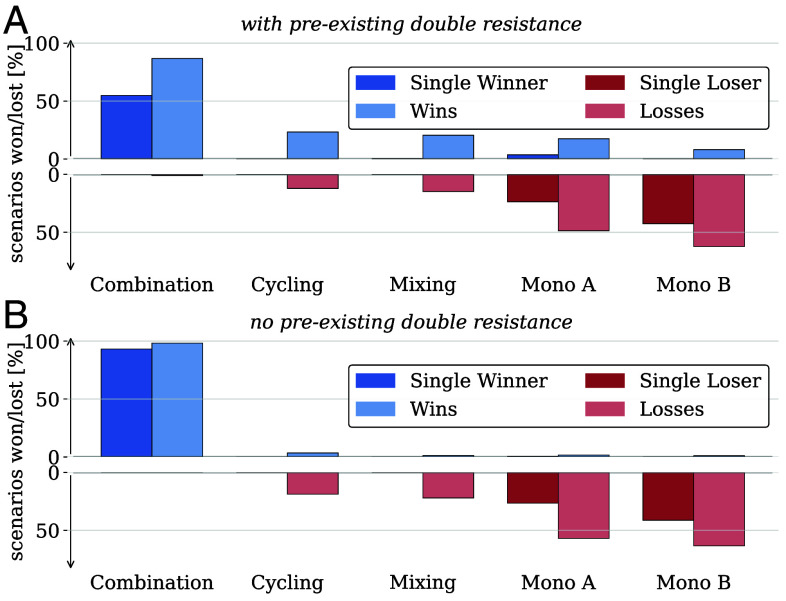
Effectiveness of the five treatment strategies in maximizing the frequency of uninfected individuals across randomly generated parameter sets. Strategies not significantly better than any other are marked as losers (pastel red), and those significantly worse than all others as single losers (dark red). Strategies not significantly worse than any other are classified as winners (pastel blue), and those significantly better than all others as single winners (dark blue). Strategies without significant differences were excluded. (*A*) 10,000 parameter sets with preexisting double resistance. 606/10,000 sets yielded no significant difference between the strategies. (*B*) 10,000 parameter sets without preexisting double resistance. 100/10,000 sets yielded no significant difference between the strategies.

In parameter sets with and without preexisting double resistance, combination therapy ranks most often as one of the best strategies (87% and 98%, respectively). It is the single best strategy in 55% of the tested parameter sets with preexisting double resistance and in 93% of cases without preexisting double resistance (Fig. 4 and *SI Appendix*, Tables S14 and S15).

In some situations (for example, when one strategy is much worse than all others), it is more important to avoid the worst strategy than selecting the very best strategy among the good ones. Our analysis finds that combination therapy is almost never among the worst strategies, while usually one of the two monotherapies performs worst. As expected, single-drug strategies perform particularly poorly when there is a high frequency of preexisting single-resistance to the applied drug (*SI Appendix*, Tables S11 and S13).

Cycling and mixing lose substantially less than the monotherapies but are rarely the single best strategy.

## Discussion

In our study, multidrug strategies, particularly combination therapy, outperformed monotherapies in reducing overall infections and the emergence of double resistance across most scenarios, while we observed most emergence of double resistance in the untreated control. Interestingly, the effectiveness of combination therapy does not stem from an increased efficacy associated with higher doses. This is because an asymmetrical antagonism exists between the bactericidal antibiotic ceftazidime (drug A) and the bacteriostatic antibiotic tetracycline (drug B), resulting in a lower clearance rate for the combination A+B compared to drug A alone (*SI Appendix*, *SI Results*). This observation implies that combination therapy may be even more advantageous when drugs are neutral or synergistic toward each other.

Why does the absence of treatment lead to worse outcomes, and why is combination therapy preventing the emergence of double resistance so effectively?

First, we measured the presence, not the density, of resistant bacteria in wells by assessing whether small aliquots of the liquid culture could grow on treated agar plates. This approach quantifies the number of wells hosting a specific resistance but can not quantify the frequency of resistance of the in-well population. The information about presence/absence alone yields important information about potential treatment success and is used in analogous clinical diagnostic methods, such as disk diffusion tests (13). We would only recognize a loss of resistance (in the experiments and clinical samples) if the resistant strain were fully outcompeted. This was not observed during the containment scenario in the untreated control. Such an outcome was expected due to the short average patient stay of 2 to 5 d in our experiments and 5 to 6 d in clinical situations (14). For the same reason, we would not expect an eradication of resistance but only a shift in resistance density, even if there were more substantial costs of resistance or higher segregational loss. In our experiments, we found no evidence of a cost of resistance (*SI Appendix*, *SI Methods*, Fig. S6 and Table S3) or segregational loss (*SI Appendix*, *SI Methods* and Table S4).

Second, in our experiments, the emergence of double resistance requires conjugation, which relies on superinfection between hosts with complementary resistance profiles. As demonstrated in Fig. 3*B*, the lowest number of superinfections occur in combination therapy, where both single-resistant strains can be cleared. Conversely, without treatment, the abundance of single resistance is highest resulting in the highest number of superinfections.

Third, the applied antibiotics affect the frequency of superinfections leading to double resistance, likely by influencing the growth dynamics within the superinfected well and potentially the conjugation rate (15). However, our experimental data are unsuitable for supporting or rejecting the impact on conjugation rates. We observed the least emergence of double resistance in superinfected wells treated with both drugs and most in untreated wells, contributing to the superiority of combination therapy and the high rates of double resistance in the absence of treatment (Fig. 3*C*). This effect on the in-well dynamics may be a property of the chosen drugs and concentrations, and we expect better results for cycling and mixing if both drugs were equally effective in suppressing double resistance or worse results for combination therapy if the combination of both drugs was less effective.

Fourth, we observed that the number of single-resistant bacteria inoculating superinfections impacts the frequency of emerging double resistance (*SI Appendix*, *SI Results* and Table S1). In our setup, superinfected wells receive two inocula, with at least one inoculum transferred from the previous plate (by infection) that has already undergone treatment for one day. When prior treatment led to a low bacterial density in the source wells, we did not observe any cases of double resistance emerging. This could magnify the effectiveness of combination therapy, where all potential single-resistant inocula transferred from the previous plate contain low bacterial densities due to effective treatment. On the one hand, this may be more a characteristic of our experimental setup due to the fixed length of the treatment interval and high clearance probabilities. On the other hand, we indeed expect fewer cases of emergence in superinfected patients if the infecting inocula are small.

In our experiments and simulations, combination therapy showed superior results in minimizing infections and preventing double resistance. This advantage may partly result from assumptions and simplifications, including the chosen strain, drugs, plasmids, and inoculum size, the discrete setup with fixed treatment durations, colonization-independent infection, and turnover probabilities, and the absence of an immune system and microbiome. Also, treating all patients irrespective of colonization diverges from clinical reality in two ways: i) In a clinical setting, some untreated patients may serve as a sanctuary for resistance and a potential source of double resistance and ii) Treating all patients, regardless of infection status, contrasts with clinical efforts to promote targeted antibiotic use. However, since patients as we model them in our in vitro experiments lack a microbiome, treating uninfecteds should have no impact on the resistance dynamics.

Despite the numerous differences between our experiments and a real clinical situation, we argue that the relative effectiveness of combination therapy in suppressing double resistance would likely translate to real patients. The reason is that the emergence of double resistance hinges on two critical processes: 1) preventing superinfections between patients carrying bacteria with complementary resistance plasmids and 2) the probability that superinfected hosts develop double resistance. We think that combination therapy offers a strategic advantage in addressing both processes.

Our results complement the findings by Angst et al. (10), who observed similar outcomes in the context of chromosomal resistance. We believe that such in vitro experimental models, which explore admittedly idealized and simplified epidemiological scenarios, can help to bridge the divide between mathematical models and randomized clinical trials. However, ultimately the evidence for or against the benefits of combination therapy must be confirmed by rigorous clinical trials with sufficient statistical power to support or challenge the effectiveness of combination therapy.

## Materials and Methods

### Drugs and Media.

In all experiments, we used LB (Sigma L3022) with 25 μg/mL (prevention scenario) or 5 μg/mL (containment and max-emergence scenario) chloramphenicol as a liquid medium and the same LB and drugs with 1.5% agar as a solid medium. Chloramphenicol was added to prevent external contaminations. We could not measure any significant growth effects of the chloramphenicol concentrations on the chloramphenicol-resistant strains (*SI Appendix*, Table S5). We used 80 μg/mL ceftazidime as drug A and 40 μg/mL tetracycline as drug B, with identical concentrations for liquid and solid media.

### Strains and Plasmids.

We used two compatible plasmids $p_{A}$ and $p_{B}$ derived from samples ESBL9 and ESBL25 from a clinical transmission study (12). Samples were kindly provided by Adrian Egli and sequenced and analyzed by Huisman et al. (11). Plasmids $p_{A}$ and $p_{B}$ provide (among other resistances) resistance against drug A and drug B, respectively. We used these plasmids and the chloramphenicol-resistant host MDS42-YFP (16) (sensitive to drugs A and B) to create three additional strains by conjugation (*SI Appendix*, Table S2) i) A-resistant, containing $p_{A}$; ii) B-resistant, containing $p_{B}$; and iii) AB-resistant, containing both plasmids (*SI Appendix*, *SI Methods*).

### Treatment Arms.

We simulated the epidemiological dynamics of six hospital wards in vitro, with each ward exploring a different treatment arm: i) control with no treatment, ii) monotherapy with ceftazidime (mono A), iii) monotherapy with tetracycline (mono B), iv) cycling therapy (A, A, B, B, …), v) mixing therapy (treatments A and B are randomly assigned daily, without knowledge of prior treatment), and vi) combination therapy (treating all patients with both drugs, each at full concentration).

### Assay Plates.

Each hospital ward was simulated in vitro on a 384-well microtiter plate (Greiner 781186). Wells are interpreted as beds in four replicate hospital wards with 94 beds each. The remaining wells contained only growth medium and remained untouched, acting as sentinels for contamination. Across all experiments and treatment arms, 2,752 control wells were used, 67 of which became contaminated. Wells with growth medium but no bacteria represent uninfected patients, whereas wells with growth medium and (resistant or sensitive) bacteria represent infected patients.

### Experimental Procedure.

Experiments were performed using a Tecan Evo 200 automated liquid handling system (Tecan) with an integrated, automated incubator (Liconic STX100, Liconic), a Tecan Infinite F200 spectrophotometer (Tecan), and a camera (Pickolo, SciRobotics).

Every day new assay plates were filled with 45 μL fresh medium and 5 μL antibiotic stock, according to its designated treatment strategy (*SI Appendix*, Fig. S1). At each of these transfers, we simulate patients staying overnight in the hospital (passage), the admission and discharge of patients (turnover), and infections between patients (infection). Passage, turnover, and infections were all done by inoculating the new plate using a pintool with retractable pins, as detailed below, carrying ≈0.3 μL drops between wells (≈1:150 dilution) leading to an approximately 6 to 8 h exponential phase. The assay plates were then incubated at 37 ^°^C and 95% relative humidity. The incubation duration varied due to variations in the time it takes to set up a new transfer and occasional transfer repetitions were made necessary because of machine errors or user mistakes. The mean incubation duration was 27 h.

We initiated the experiment by inoculating one 384-well plate from fresh overnight cultures representing patients from an outside community. We assume that this community is sufficiently large to be unaffected by interactions with the hospital ward. Incoming patients are either uninfected or carry one of the four strains (sensitive, each single resistant or double resistant) and are sampled according to predefined sampling proportions, defining the probability of a resistance profile being admitted to the hospital (Table 1). This initial plate remained untreated and was used as the initial population for all six treatment arms.

### Turnover.

Each patient has a turnover probability τ to be discharged from the hospital and replaced by a newly admitted patient during a transfer. Wells representing staying patients were passed from the previous to the new assay plate using the pintool. Here, the pins for discharged patients are retracted. Vacant beds on the assay plate were then reoccupied by patients from the community analogous to the initial setup.

### Infections.

To simulate infections, each well has an infection probability β to infect another randomly chosen well on the next assay plate during the transfer. Therefore, each patient can infect at most one other patient per transfer, but several patients could potentially infect the same patient.

### Resistance Profiles.

To assess the resistance profile of each well, we spotted the previous assay plate onto four agar plates, using the pintool. Three plates were treated with antibiotics (A, B, or AB), while one was untreated (none). After incubation at 37 ^°^ C and 95% relative humidity, images of the agar plates are taken and analyzed using the Pickolo package (SciRobotics, Kfar Saba, Israel). The software automatically detects the presence of colonies at each well position, which we also manually verified. The growth pattern on the four agar plates allowed us to determine the resistance profile of a well, which reflects how the well would behave if treated.

By default, we distinguish six resistance profiles (*SI Appendix*, Table S6). The wells may either be 1) uninfected (U), 2) exclusively infected with sensitive bacteria (S), 3) infected with A-resistant bacteria ($A_{r}$), 4) infected with B-resistant bacteria ($B_{r}$), 5) infected with AB-resistant bacteria $\left(AB_{r}\right)$, 6) or be infected with a mixed population containing A-resistant and B-resistant bacteria, but no AB-resistant bacteria $\left({\left(A\&B\right)}_{r}\right)$. The way we classify the resistance profiles of the bacterial population in a well leads to the dominance of resistance, in the sense that a predominantly sensitive population harboring a resistant minority would be classified as resistant (*SI Appendix*, Table S7). Any observed growth pattern not corresponding to the six resistance profiles mentioned above is classified as *other*. The resistance profile other primarily occurs when bacterial densities are low (see also *SI Appendix*, *SI Methods*).

### Scenarios.

We conducted experiments for three scenarios (prevention, containment, and maximum-emergence) with 14 to 27 transfers each. Each experiment was defined by a different parameter set consisting of i) the infection probability β within the hospital, ii) the turnover probability τ, and iii) the sampling proportions $c_{\phi }$ of patients with resistance profile $\phi \in \{U,S,A_{r},B_{r},AB_{r}\}$ (Table 1).

The prevention scenario (*SI Appendix*, Fig. S2) addresses how the treatment strategies perform with a moderately resistant community and a moderate infection regime in the hospital ward and how well they are able to prevent the upcoming double resistance.

The containment scenario (*SI Appendix*, Fig. S4) corresponds to a scenario in which some patients entering the hospital are infected with double-resistant bacteria to compare the ability of treatment strategies to contain the spread of preexisting double resistance.

During the maximum-emergence scenario (Fig. 2) 50 % of the incoming patients are infected with A-resistant bacteria, and the other 50 % are infected with B-resistant bacteria. These conditions maximally favor opportunities for horizontal gene transfer. The basic reproduction number was set to $R_{0}=0.5$ (*SI Appendix*, Eq. **S1**) to ensure that double-resistant strains are flushed out, reducing the stochastic dependency on earlier emergence events while maintaining a high potential for new emergence.

### Instruction Sets.

Based on the parameter defined for each experiment (Table 1), we generated instructions that were passed to the liquid handling platform. These instructions specify which patients are passaged or discharged and admitted, who infects whom, and the treatment for mixing therapy. Instructions are randomly generated prior to each transfer. We call the entirety of all instructions that come up during an experimental run an instruction set. Instruction sets are identical across all treatment arms and replicates.

### Computational Model.

We created a stochastic model (*SI Appendix*, *SI Computational Model*) incorporating 94 in silico patients, each capable of adopting one of six resistance profiles $\phi \in \{U,S,A_{r},B_{r},AB_{r},\left(A_{r}\&B_{r}\right)\}$. The model is structured analogue to the in vitro experiments (*SI Appendix*, Fig. S1) and alternates between modeling the transactions between wells and the effect of treatment during incubation.

Admission and discharge (turnover) were simulated by replacing the resistance profile of the current patient with that of the incoming patient, as defined by the instruction set. Infections are simulated by combining the resistance profiles of the receiving well i and the infecting well j. The resulting resistance profile $\phi_{i}+\phi_{j}$ is determined using the rules based on the dominance of resistance specified in *SI Appendix*, Table S9. Calculations involving more than two resistance profiles apply the associative law and are determined pairwise, e.g., $\left(U+S\right)+A_{r}=S+A_{r}=A_{r}$.

To model treatment effects, we use transition probabilities to assign the postincubation resistance profile $\phi (\hat{T})$ stochastically based on the treatment and the preincubation resistance profile ϕ(T). The transition probabilities (*SI Appendix*, Tables S18–S25) were estimated based on experimental data across all experiments.

### In Silico Sensitivity Analysis.

To augment the experimental data, we conducted an in silico sensitivity analysis. We randomly generated 10,000 parameter sets with and 10,000 without preexisting double resistance. Turnover and infection probabilities were uniformly sampled [0.05,0.95], allowing for $R_{0}\in \left[0.0526,19\right]$. The sampling proportions $c_{\phi }$ for all incoming resistance profiles ($\phi \in \{U,S,A_{r},B_{r},AB_{r}\}$) were randomized by sampling a number $n_{\phi }\in \left[0,1\right]$ from a uniform distribution and subsequently normalizing by the sum: $c_{\phi }=n_{\phi }/\sum_{j}n_{j}$. We created ten randomized instruction sets for each parameter set and conducted one simulation per instruction set (*SI Appendix*, Fig. S3*D*) for 28 transfers.

For this analysis, the frequency of noninfected individuals during the last four transfers was used as a performance metric for treatment strategies, as it also indirectly reflects the frequency of both double- and single-resistant patients. We conducted an ANOVA test to assess whether the effect of the treatment strategies significantly (P<0.05) influences the frequency of uninfecteds. For significant tests, we proceeded with Tukey’s post hoc analysis (P<0.05), identifying significantly distinct pairs of strategies. Strategies not significantly inferior to others were classified as winners, while strategies not significantly superior to any were classified as losers. Strategies that win or lose a parameter set alone are single winners or single losers.

## Supplementary Material

Appendix 01 (PDF)

## Data Availability

Experimental data and analysis scripts and code for the computational model data have been deposited in Zenodo (17). Some study data are available. (Simulation results are available on request due to large file size. Source code to run simulations is included above.)

## References

[1] P. F. Kable, Selection for tolerance in organisms exposed to sprays of biocide mixtures: A theoretical model. Phytopathology **70**, 8 (1980).

[2] C. J. Delp, Coping with resistance to plant disease control agents. Plant Dis. **64**, 652–657 (1980).

[3] G. Skylakakis, Effects of alternating and mixing pesticides on the buildup of fungal resistance. Phytopathology **71**, 1119–1121 (1981).

[4] D. E. Goldberg, R. F. Siliciano, W. R. Jacobs, Outwitting evolution: Fighting drug-resistant TB, Malaria, and HIV. Cell **148**, 1271–1283 (2012). 22424234 10.1016/j.cell.2012.02.021 PMC3322542

[5] B. Siedentop , The effect of combining antibiotics on resistance: A systematic review and meta-analysis. eLife **13**, RP93740 (2024). 39704726 10.7554/eLife.93740 PMC11661791

[6] P. J. van Duijn , The effects of antibiotic cycling and mixing on antibiotic resistance in intensive care units: A cluster-randomised crossover trial. Lancet Infect. Dis. **18**, 401–409 (2018). 29396000 10.1016/S1473-3099(18)30056-2

[7] H. Uecker, S. Bonhoeffer, Antibiotic treatment protocols revisited: The challenges of a conclusive assessment by mathematical modelling. J. R. Soc. Interface **18**, 20210308 (2021). 34428945 10.1098/rsif.2021.0308 PMC8385374

[8] S. Bonhoeffer, M. Lipsitch, B. R. Levin, Evaluating treatment protocols to prevent antibiotic resistance. Proc. Natl. Acad. Sci. U.S.A. **94**, 12106–12111 (1997). 9342370 10.1073/pnas.94.22.12106 PMC23718

[9] B. Tepekule, H. Uecker, I. Derungs, A. Frenoy, S. Bonhoeffer, Modeling antibiotic treatment in hospitals: A systematic approach shows benefits of combination therapy over cycling, mixing, and mono-drug therapies. PLoS Comput. Biol. **13**, 1–22 (2017). 10.1371/journal.pcbi.1005745 PMC5600366 28915236

[10] D. C. Angst, B. Tepekule, L. Sun, B. Bogos, S. Bonhoeffer, Comparing treatment strategies to reduce antibiotic resistance in an in vitro epidemiological setting. Proc. Natl. Acad. Sci. U.S.A. **118**, 1–7 (2021). 10.1073/pnas.2023467118 PMC8020770 33766914

[11] J. S. Huisman , The effect of sequencing and assembly on the inference of horizontal gene transfer on chromosomal and plasmid phylogenies. Philos. Trans. R. Soc. B Biol. Sci. **377**, 20210245 (2022). 10.1098/rstb.2021.0245 PMC9393563 35989605

[12] S. Tschudin-Sutter , Prospective validation of cessation of contact precautions for extended-spectrum *β*-lactamase-producing *Escherichia coli*. Emerg. Infect. Dis. **22**, 1094–1097 (2016). 27191171 10.3201/eid2206.150554 PMC4880108

[13] The European Committee on Antimicrobial Susceptibility Testing, Antimicrobial susceptibility testing EUCAST disk diffusion method Version 12.0. EUCAST. http://www.eucast.org. Accessed 29 November 2024.

[14] K. Hofstetter, E. Salgado-Thalmann, M. Bachmann, Kennzahlen der Schweizer Spitäler 2015. https://www.bag.admin.ch. Accessed 29 November 2024.

[15] B. Headd, S. A. Bradford, Physicochemical factors that favor conjugation of an antibiotic resistant plasmid in non-growing bacterial cultures in the absence and presence of antibiotics. Front. Microbiol. **9**, 1–14 (2018). 30254617 10.3389/fmicb.2018.02122 PMC6141735

[16] T. Fehér , Competition between transposable elements and mutator genes in bacteria. Mol. Biol. Evol. **29**, 3153–3159 (2012). 22527906 10.1093/molbev/mss122 PMC3457772

[17] M. Muetter, mmuetter/tsplasmid: Revision. Zenodo. 10.5281/zenodo.14137410. Deposited 13 November 2024.

