# Supplementary material for: The impact of treatment strategies on the epidemiological dynamics of plasmid-conferred antibiotic resistance
Source: Proc Natl Acad Sci U S A. 2024 Dec 17;121(52):e2406818121. doi: 10.1073/pnas.2406818121 (PMC11670247; doi:10.1073/pnas.2406818121)
Supplement: Supplementary file 1 — Appendix 01 (PDF) [file pnas.2406818121.sapp.pdf]

Supporting Information for

The Impact of Treatment Strategies on the Epidemiological Dynamics of Plasmid-Conferred Antibiotic Resistance

Malte Muetter, Daniel C. Angst, Roland R. Regoes, Sebastian Bonhoeffer

[2]To whom correspondence should be addressed. E-mail: malte.muetter@env.ethz.ch

This PDF file includes:

Supporting text
Figs. S1 to S8
Tables S1 to S56
SI References

Supporting Information Text

A. SI Methods.

Strains We chose two plasmid-carrying donors, ESBL9 and ESBL25, and two drugs, ceftazidime and tetracycline, based on the resistance conferred by the plasmids contained in the strains and the compatibility of the plasmids. ESBL9 and ESBL25 were collected as part of a clinical transmission study at the University Hospital Basel, Switzerland (1) and fully sequenced, including identification of the carried plasmids (2). The strains were a generous gift from Adrian Egli, University Hospital Basel.

ESBL9 contains an IncI1 plasmid, referred to here as p_A, conferring, among others, resistance to ampicillin and ceftazidime but not tetracycline or chloramphenicol. ESBL25 contains an IncF1 plasmid, referred to here as p_B, conferring, among others, resistance to ampicillin and tetracycline but not ceftazidime or chloramphenicol.

The two plasmids were transferred by conjugation from the original clinical isolates to the chloramphenicol-resistant and ampicillin-sensitive *Escherichia coli* MDS42-YFP (recipient) (3), followed by selection for ampicillin and chloramphenicol resistance. This results in the ceftazidime-resistant (A-resistant) strain and the tetracycline-resistant (B-resistant) strain. The double-resistant (AB-resistant) strain was created by a further round of conjugation to receive both plasmids and subsequent selection for ceftazidime and tetracycline resistance. Strains are listed in Table S2. All transconjugants were verified by PCR replicon typing using primers specific for the respective replicon (4).

Drugs. We used ceftazidime, referred to as drug A, at a concentration of 80 µg/ml. 80 µg/ml is substantially lower than the MIC for A-resistant bacteria and more than 50 times the MIC for sensitive or B-resistant bacteria. Using the same reasoning, we used tetracycline, referred to as drug B, at a concentration 40 µg/ml. The antibiotic concentrations in the liquid and the solid media were identical. To avoid contamination, we used 25 µg/ml chloramphenicol for *prevention* scenario and 5 µg/ml chloramphenicol for *containment* and *maximum-emergence* scenarios. We could not measure any significant growth effects of the chloramphenicol concentrations on the chloramphenicol-resistant strains (see Table S5).

Conjugation Protocol. We used ampicillin-resistant and chloramphenicol-sensitive original donors (1, 5) and the chloramphenicol-resistant, ampicillin-sensitive recipient (3). Fresh overnight cultures of both donors and recipients were diluted 1:1000 and grown to mid-exponential phase. Following this, the donor and recipient cultures were combined in a culture tube and incubated for four hours at 37 °C with constant shaking at 180 rpm. We then spotted a 100 µL drop of this mixture on an agar plate treated with 25 $\frac{\mu g}{mL}$ chloramphenicol and 100 $\frac{\mu g}{mL}$ ampicillin, allowing only the transconjugants to grow. Conjugation was verified by PCR replicon typing (4).

Plasmid costs To measure plasmid costs, we grew three replicates of overnight cultures of all strains in selective medium. The cultures were then diluted approximately 1:150 into LB with 5 $\mu g/ml$ chloramphenicol using the pintool, following the same procedure as in the main experiments. Subsequently, we recorded OD growth curves using the same plate reader. The maximum growth rate was estimated by applying linear regression to a sliding window on the log-transformed data (window size: 1 hour, step size: approximately 5 minutes). Pairwise comparisons were performed between the maximum growth rates of the sensitive strain and the plasmid-carrying strains using the Mann-Whitney U test (scipy.stats (6)), followed by a Bonferroni correction to account for multiple testing. We observed no significant difference in the maximum growth rate between any of the pairs (Table S3).

Segregational Loss We estimated plasmid segregation loss over 24 hours (t_0–t_1) without treatment and with treatment as a control. For this, we grew overnight cultures in selective medium for three replicates $k \in \{1, 2, 3\}$ of each plasmid-carrying strain. We diluted the cultures and plated each on drug-free agar plates, followed by replica plating onto selective plates to identify the presence or absence of resistance plasmids in each colony. This initial step represents time point t_0. The overnight cultures were then transferred to i) drug-free medium and ii) selective medium (control), using the same pintool as in the main experiments. The cultures were incubated for 24 hours, after which we diluted and plated them again on drug-free plates and used replica plating on selective plates to assess plasmid presence for time point t_1. We compared the frequencies $f_k(t)$ of plasmid presence between time points t_0 and t_1 using the Mann-Whitney U test (scipy.stats (6)). No significant loss of plasmids was observed in either the control or the experimental conditions (Table S4). We estimated the mean frequency of plasmid presence $f(t)$ for each strain and time point and the confidence intervals $CI(t)$ for the frequency by bootstrapping the pooled colony presence-absence data.

Growth rates and bacterial density. We assessed the final bacterial density of overnight cultures following an 18-hour incubation period (Table S5) for each bacterial strain in its respective selective medium for two different chloramphenicol concentrations: 5 $\frac{\mu g}{mL}$ and 25 $\frac{\mu g}{mL}$. To estimate the bacterial density, we plated 200 µL of various dilutions of the cultures on agar plates using glass beads. The 95 % confidence intervals for the colony counts were calculated using the Poisson distribution.

In addition, we monitored the optical density (OD) in 384-well plates over an 18-hour period in the respective selective medium containing either 5 $\frac{\mu g}{mL}$ or 25 $\frac{\mu g}{mL}$ chloramphenicol. To determine the maximum growth rates, we employed a sliding window approach with a one-hour width, linearly fitting the growth rate to the log transformed values within this window for each replicate. The 95 % confidence intervals for the maximal growth rates were calculated using the Student's t-distribution.

Basic Reproductive Number. R_0 represents the number of secondary infections generated by one patient in a fully susceptible population. Let τ denote the probability that a patient leaves the hospital ward after one timestep, $\tau' = 1 - \tau$ the probability

that the patient stays and β the probability that a patient infects another during one timestep. Then we can write the total number of infections caused by one patient introduced into a susceptible population as a geometric series:

$$R_0 = (\tau')^0 \cdot \beta + (\tau')^1 \cdot \beta + (\tau')^2 \cdot \beta + ... = \beta \sum_{n=0}^{\infty} (\tau')^n$$

This geometric series can then be rewritten as:

$$R_0 = \beta \cdot \frac{1}{1-\tau'} = \frac{\beta}{\tau} \quad \text{[S1]}$$

Phenotyping – Limitations. The phenotyping procedure enables high throughput identification of resistance profiles. Although this method is generally reliable and effective for most wells, it is difficult to accurately determine the resistance profile for wells with very low bacterial densities due to the potential for stochastic effects. We analysed 1784 A_r turnover wells treated with antibiotic B during the *maximum-emergence* scenario. Here, we observed, alongside the expected A_r and U wells, 9% 'other' and 17% S wells, as detailed in Figure S5A. It might be tempting to interpret the S wells as those in which all bacteria lost their plasmids and the *'other'* wells as artefacts of measurement errors. Although these interpretations are not incorrect, a more critical factor influencing the measured resistance profile distribution is the inherent stochasticity of the method if applied to low-density wells.

As an example, we will analyse wells identified as A_r-wells during the previous transfer and subsequently treated with antibiotic B. To simplify the following analysis, we only consider *agar plates* treated with drug $\vartheta \in \{N, A\}$ (no drug, drug A) and disregard plates treated with drug B (B-plates) and AB (AB-plates). Furthermore, we will use a prime (') to indicate a counter probability ($w' = 1 - w$). Drawing a drop with volume V_{drop} from a well with volume V_{well} leads to a probability $p = \frac{V_{\text{drop}}}{V_{\text{well}}}$ of drawing a specific bacterium. The probability that the drawn drop contains no bacteria of phenotype $\psi \in \{\emptyset, a\}$ (without resistance and A-resistance) is $d'_\psi = (1-p)^{Z_\psi}$, with Z_ψ representing the number of bacteria with phenotype ψ inside the well. We denote the probability that a drop can grow on a plate treated with drug ϑ as g_ϑ. The probability g'_N of drawing a drop that will not form a colony on an N-plate can than be defined as $g'_N = d'_\emptyset d'_a$, and the probability g'_A of drawing a drop that will not grow on an A-plate is $g'_A = d'_a$.

Assuming that the drawn drop does not significantly alter the well volume and composition, we obtain the following probabilities for the possible resistance profiles (see also Figure S5B):

$$\mathbb{P}(U) = g'_N \cdot g'_A$$
$$\mathbb{P}(S) = g_N \cdot g'_A$$
$$\mathbb{P}(A_r) = g_N \cdot g_A$$
$$\mathbb{P}(other) = g'_N \cdot g_A$$

Assuming $p = 0.006$ (value for our experiment), a well containing 25 A-resistant and 15 sensitive bacteria will result in 70% U, 26% S, 3% A_r, and 11% *other*. This result is similar to the experimentally measured distribution (compare Figure S5A).

Advanced Resistance Profiles We introduced advanced phenotypes into our analysis to determine the approximate bacterial density inside the wells. Wells that retain their phenotype after one transfer, despite being sensitive to a particular antibiotic, are expected to exhibit a low bacterial density post-treatment and are labelled ϕ^l. Conversely, wells that were either untreated or treated with an ineffective antibiotic are expected to contain a high bacterial density and are labelled ϕ^h. In addition, wells that underwent a change in resistance profile or were mixed with other wells are denoted as $\phi^?$ and are excluded from further analysis. We summarised the influence of the bacterial densities within the inoculating wells on the frequency of superinfections developing double resistance in Table S1.

Statistical Analysis. To compare the performance of different treatment strategies *in vitro*, we summarized the resistance profiles into groups and focused our analysis on three groups: uninfected, single-resistant and double-resistant. These labels stem from the properties within the wells. That means single resistant wells (A_r, B_r or (A_r&B_r) would be wells that contain only single resistant (or sensitive) bacteria, but no double resistant bacteria and therefore can be cured by combining drugs. In contrast, double-resistant wells cannot be cured using both antibiotics simultaneously since they contain AB-resistant bacteria. We then averaged the frequency of each group over the last four transfers for every replicate. Four transfers correspond to a complete cycle in the cycling strategy with a period of 2 (e.g., A-A-B-B). The effect of the treatment strategy on the average frequencies of uninfected, single-resistant, and double-resistant wells was then tested using a one-way ANOVA. In the case of a significant test ($p < 0.05$), we conducted a pairwise Tukey post hoc analysis to relate the mean frequencies.

In addition, we analysed superinfections between patients and the emergence of double resistance across different strategies in the *maximum-emergence* scenario. We considered all measurement points from the fourth transfer onwards as at near-stationary level for the non-cycling strategies. Consequently, the initial conditions for each new transfer remain approximately the same or are repeated every fourth transfer in the case of cycling.

For this analysis, we counted the number of newly emerged double-resistant wells $n_\mathcal{E}$ and the number of superinfections $n_\mathcal{S}$ across all replicates for each plate, with each plate representing one treatment arm for a single transfer. Newly emerged double-resistant wells are defined as those exhibiting double resistance but not having previously been passaged or infected by

a double-resistant well. Additionally, for each plate, we analysed all wells treated with treatment ϑ, counting the number of superinfected wells $n_{\mathcal{S}}^{\vartheta}$ and those among them that developed double resistance $n_{\mathcal{E}}^{\vartheta}$.

We then tested whether the treatment strategy significantly affects the emergence frequency $f_{\mathcal{E}} = n_{\mathcal{E}}/n_P$ and the frequency of superinfection $f_{\mathcal{S}} = n_{\mathcal{S}}/n_P$, with n_P being the number of patients in a hospital ward across all replicates (376) using an ANOVA. Additionally, we used an ANOVA to assess if treatment ϑ significantly influences the frequency of superinfected wells that develop double resistance $n_{\mathcal{E}}^{\vartheta}/n_{\mathcal{S}}^{\vartheta}$.

Subsequent to a significant ANOVA test, we conducted pairwise Tukey post hoc comparisons between the treatment arms ($p < 0.05$).

All statistical analyses were performed in Python 3.8.5 using SciPy's f_oneway() (6) for ANOVA tests and Statsmodels' pairwise_tukeyhsd() (7) for conducting Tukey's honest significant difference post hoc analyses.

***Maximum-emergence* scenario: Predicting the Emergence Probability.** We counted for each plate i the number of superinfected wells $n_{\mathcal{S}}^{i}$ and normalized them by the number of patients per plate n_p to calculate the frequency of superinfection ($f_{\mathcal{S}}^{i} = \frac{n_{\mathcal{S}}^{i}}{n_p}$). Then, we estimated the probability of superinfection $\mathbb{P}(\mathcal{S})$ for each treatment arm by averaging $f_{\mathcal{S}}^{i}$. In addition, we approximated the probability of emergence for superinfected wells $\mathbb{P}_{\vartheta}(\mathcal{E}|\mathcal{S})$ under treatment ϑ, by normalizing the total number of newly emerged resistances across all plates $N_{\mathcal{E}}^{\vartheta}$ by the total number of superinfected wells under treatment ϑ ($N_{\mathcal{S}}^{\vartheta}$): $\frac{N_{\mathcal{E}}^{\vartheta}}{N_{\mathcal{S}}^{\vartheta}}$. The estimates for $\mathbb{P}_{\vartheta}(\mathcal{E}|\mathcal{S})$ were then utilized to approximate the average probability of superinfected wells developing double resistance for each treatment arm. The weighted average of all $\mathbb{P}_{\vartheta}(\mathcal{E}|\mathcal{S})$ were computed using the proportion of patients receiving treatment ϑ as weights. For example, in mixing, the average probability $\overline{\mathbb{P}}(\mathcal{E}|\mathcal{S})$ is given by $0.5\mathbb{P}_A(\mathcal{E}|\mathcal{S}) + 0.5\mathbb{P}_B(\mathcal{E}|\mathcal{S})$. We then used Equation S2 to predict the average probability of emerging double resistance $\mathbb{P}(\mathcal{E})$ for each strategy, as indicated in Figure 3A by black stars.

$$\mathbb{P}(\mathcal{E}) = \mathbb{P}(\mathcal{S})\overline{\mathbb{P}}(\mathcal{E}|\mathcal{S}) \qquad [S2]$$

B. SI Results.

Impact of Treatment on the Emergence of Double Resistance. As demonstrated in Figure 3B, our findings indicate that treatment substantially influences the frequency of emerging double resistance in superinfected wells. Population dynamics within wells can potentially explain these results. Here we approximate $\mathbb{E}_{\vartheta}$, the expected number of conjugations during one treatment phase under treatment ϑ, as $\mathbb{E}_{\vartheta} \propto \gamma_{\vartheta} \int_{t1}^{t2} {}^{A}X_{\vartheta}(t)\, {}^{B}X_{\vartheta}(t)dt$. ${}^{i}X$ represents the density of bacteria with resistance i, and γ_{ϑ} is the treatment dependent conjugation rate. The experimentally generated data are insufficient for adequately estimating γ_{ϑ}. However, if we assume identical initial bacterial populations, we can qualitatively rank the cumulative product of bacterial densities $\int_{t1}^{t2} {}^{A}X_{\vartheta}(t)\, {}^{B}X_{\vartheta}(t)dt$. The highest cumulative product is achieved when ${}^{A}X$ and ${}^{B}X$ grow without or with ineffective treatment. Additionally, we know that the clearance rate of ceftazidime (drug A) is substantially higher than that of tetracycline (drug B), as shown in Table S8, resulting in a larger cumulative product over time when treated with antibiotic B. Lastly, the lowest cumulative product is associated with treatment AB, where neither of the two strains can grow. Therefore, if we disregard γ_{ϑ}, the above reasoning predicts the following ranking for the number of emergences per superinfection: None, B, A, AB. This predicted ranking aligns with the ranking observed in Figure 3C.

Another potential explanation are potential differences in the plasmid-specific conjugation rates. If, for example, p_B had a higher conjugate rate than p_A, then drug A would have a stronger impact on the emergence of double resistance, even if we assumed identical clearance rates.

Treatment strategies influence the number of bacteria inoculating superinfections. We observed that the number of single-resistant bacteria that inoculate superinfections affects the emergence of double resistance (Table S1). At least one of the two superinfection-initiating inocula originates from infections between patients and is sourced from the previous assay plate. The cell densities and compositions within these source wells, which have already undergone treatment for one day, vary considerably depending on the resistance profile ϕ and the treatment history. We used 'advanced phenotypes' (see SI Methods) to distinguish between high-density (ϕ^h) and low-density (ϕ^l) wells, assigning these based on the wells resistance profile (ϕ) and treatment history.

During the *maximum-emergence* scenario, which contained the highest number of superinfections, we made two noteworthy observations. First, superinfections between ϕ^h and ϕ^l ($A_r^l + B_r^h$ (43 superinfections) and $A_r^h + B_r^l$ (one superinfection)) occur significantly less frequently than superinfections between $A_r^h + B_r^h$ (a total of 1176 superinfections, as outlined in Table S1). This discrepancy can be attributed to the high clearance rates of both drugs, resulting in a higher prevalence of ϕ^h compared to ϕ^l.

Second, none of the 44 superinfections involving $A_r^l + B_r^h$ and $A_r^h + B_r^l$ resulted in double resistance.

Antagonism. Adding the bacteriostatic antibiotic tetracycline (drug B) reduces the probability of clearing sensitive bacteria with the bactericidal antibiotic ceftazidime (drug A). The clearance probability drops from 0.97 to 0.86, as shown in Table S8. Antagonism between bactericidal and bacteriostatic antibiotics has been documented by various researchers since the 1950s, as exemplified by the works of Cates (8) and Jawetz (9), and also more recently by Ocampo (10). The antagonistic effect may arise because the bacteriostatic drug (tetracycline) lowers the growth rate, resulting in a decreased kill rate of the bactericidal drug (ceftazidime) (11). Accordingly, this antagonistic effect is anticipated to be less pronounced for a tetracycline-resistant

strain, where the impact on the growth rate is diminished. This hypothesis is supported by the measured clearance rates for B_r wells, where the clearance probability remains at 0.98 for treatment with drugs A and AB Table S8.

C. SI Computational Model.

Stochasticity We conducted three experiments, each defined by one parameter set consisting of a turnover probability τ, an infection probability β and the probability distribution for sampling patients c_ϕ with different resistance profiles $\phi \in \{U, S, A_r, B_r, AB_r\}$. For each experiment, we randomly generated one instruction set based on the given parameter set. Due to the scale and complexity of the experiment, it was infeasible to conduct a unique instruction set for each replicate. Therefore, we opted to employ identical instruction sets for all replicates, which reduces the number of robot arm movements (and time) for infection and turnover by a factor of four.

As a consequence, our replicates may be interpreted as patients with identical histories regarding original infection, treatment and exchange with other patients. However, due to the accumulated biological stochasticity along the patient histories, their phenotypic properties may vary, as reflected by the variance of the replicates.

Since we only tested one instruction set per replicate *in vitro*, we wondered whether the measured results depend on the randomisation of the instruction set and if we would expect a qualitatively different result if we reran the experiment 100 times. To answer this question, we created a computational model that, for a given parameter set, rerandomises the instruction set and conducts a stochastic simulation to mimic the biological variability. In Figure S3, we visualised the different sources of experimental and computational variability.

Transition Probabilities. We used the experimental data to calculate the transition frequencies for all pre-treatment $\phi^i_\vartheta(T)$ to post-treatment $\phi^i_\vartheta(\hat{T})$ resistance profile combinations for each plate i and treatment ϑ. $\phi(\hat{T})$ is measured during the phenotyping procedure, while $\phi(T)$ is estimated by employing one plate-handling simulation step to $\phi(\hat{T}-1)$ as described in the methods (e.g. $A_r + S \rightarrow A_r$). Then, we estimated the transition probabilities as the weighted average of transition frequencies across all plates, with the count of $\phi_\vartheta(T)$ on each plate as a weight.

For each treatment ϑ, we created one transition matrix M^ϑ, with the pre-treatment resistance profile $\phi(T)$ as columns and the post-treatment profile $\phi(\hat{T})$ in the rows (Table S18–S21). To simulate the incubation phase, we use M^ϑ to stochastically assign the post-treatment resistance profile $\phi_\vartheta(\hat{T})$, using the respective column of the transition matrix as a probability distribution.

Transition Probabilities for Transfer 1. All patients are untreated during transfer 0, leading to exceptionally high rates of superinfections and high emergence rates per superinfection during transfer 1. To account for this, we created four additional transition matrices for simulating the first transfer (see Table S22–S25).

Choice of Model. We also considered using a continuous model. However, a typical population-based model would not match the experimental measurements for effectively treated patients. This is due to the discrete nature of our experimental setup. Here, the frequency of infected patients has a local maximum before treatment and a local minimum after treatment, creating sawtooth-shaped frequencies over time. We conducted the phenotyping at the end of the incubation period, at the local low point, diverging from the average frequencies predicted by a continuous model. Therefore, a continuous model would either use realistic clearance rates and not fit through the experimental data points or use exaggerated clearance rates and fit through the data points. For example, treating an S well with a hypothetical drug C leads to a steady decline in bacterial density over time, resulting in a bacterial density below the detection limit after 24 hours (our first experimental measurement point). If initially, all wells are infected, and at the first measurement point, the infection drops to 0%, fitting a continuous compartmental model to these data would result in infinite clearance rates.

Contamination of the Transition Matrix. The computational simulations employ four transition matrices ($M^{\text{none}}, M^A, M^B, M^{AB}$) derived from the observed transitions during the three experiments. During the experiments, we observed a low rate of contamination affecting neighbouring wells, likely due to pintool and plate movements by the liquid handling platform. Quantifying the exact contamination rate is challenging, though the observed mean transition probability from U to U is 0.99%.

These contaminations can be inconsequential; for example, an S well contaminating an A_r well will not cause a shift of the resistance profile in the contaminated well. However, they can also lead to artefactual transitions that are reflected in the transition matrices, such as $U \rightarrow S$ (Table S18), $U \rightarrow A_r$ (Table S19), or $U \rightarrow B_r$ (Table S20). The impact of the recorded artefactual transitions in the transition matrices depends on the frequency and the transferred resistance profile. For instance, in Mono A, a high frequency of A_r contaminations is observed due to the predominant presence of A_r wells, creating the impossible transition $U \rightarrow A_r$ described above, which now occurs in all simulations independent of the presence or frequency of A_r during the simulation. Similarly, in the *containment* experiment, the abundance of AB_r wells in all treatment arms led to a higher rate of double-resistant contamination reflected in the transitions: $A_r \rightarrow AB_r$ (Table S18) and $A_r \rightarrow AB_r$ & $B_r \rightarrow AB_r$ (Table S20). Resistance mutations could also explain these transitions; however, because they occurred mainly during the *containment* scenario and the fact that they exclusively came up in wells neighbouring double-resistant wells, we believe that they are an artefact of unintended infections.

Artefactual transitions such as $U \rightarrow S$, or $U \rightarrow A_r$ have a neglectable effect on the simulation of all scenarios, as their occurrence in the regular infection and admission processes outweighs the contribution through the artefactual transitions. Similarly, double-resistant contaminations minimally impact the simulations of both the *containment* scenario (where double resistance is regularly admitted) and the *maximum-emergence* scenario (due to a low R_0 and frequent emergence of double

resistance). However, they pose a problem to the simulation of *prevention* scenario, where a low (untruthful) influx of AB_r can spread ($R_0 > 1$).

Filtered Transition Probabilities. To mitigate the effect of contaminated transition matrices, we introduced filtered transition matrices. For this, we assumed no resistance mutations and forbidding impossible transitions (by setting the transition probability $U \rightarrow U$ to 1; see Table S26 – S33).

Using these filtered probabilities to simulate the *prevention* scenario leads to better-matching results, almost removing the spread of double resistance in Mono A and the multidrug strategies and thereby matching the experimental data better (compare the green error bands between Figure S2 & Figure S7). In addition, we conducted a secondary sensitivity analysis with these filtered probabilities (see Figure S8). Because the overall conclusions are consistent between the simulations using filtered and unfiltered transition probabilities, we opted to use the unfiltered transition probabilities in the main paper for a more direct representation of the experimental data.

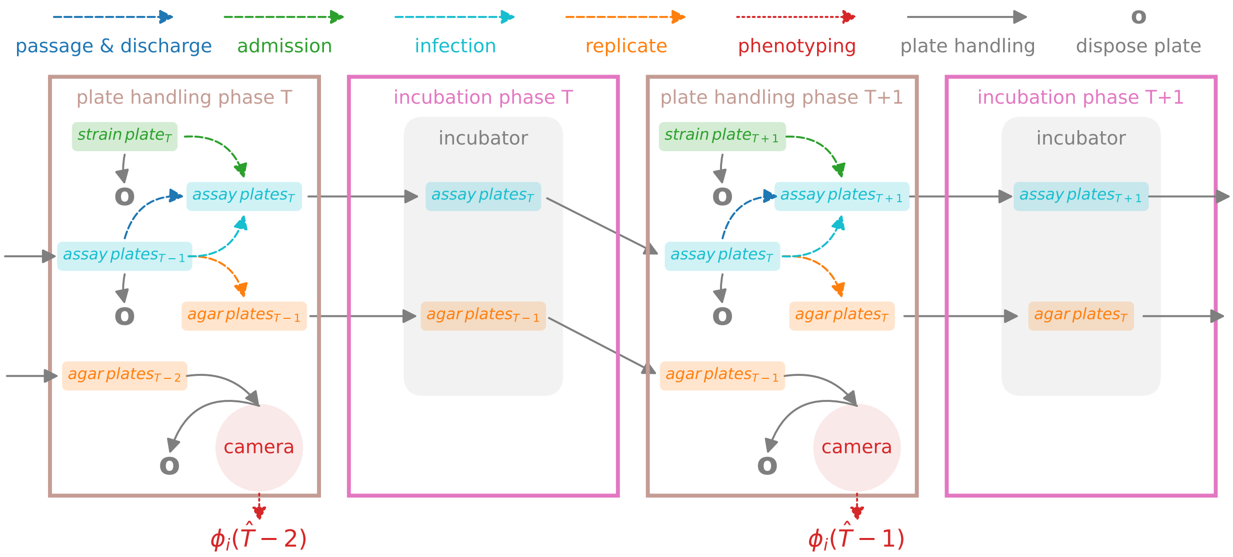

Fig. S1. Schematic illustrating the procedure used in the experiment for transfers T and T+1 in the liquid handling platform after adding medium and drugs to the *assay plates*. Every transfer (day), we provide new *assay* and *agar plates*. Plates from the previous transfers are removed. To inoculate the new *assay plates* with newly admitted patients from the strain plate, along with staying patients and infection between patients from the previous *assay plate*, we use a pintool with retractable pins (dashed lines). Discharged patients are not transferred (pins retracted) to the new assay plates. Plates are then automatically transferred (solid lines) to the incubator for overnight incubation. Subsequently, we replicate each *assay plate* onto four agar plates using the pintool. These plates are treated with antibiotics A, B, and AB, and one remains untreated. Once the agar plates have been incubated overnight, we capture images (dotted lines) to determine the resistance profile ϕ_i for each well i.

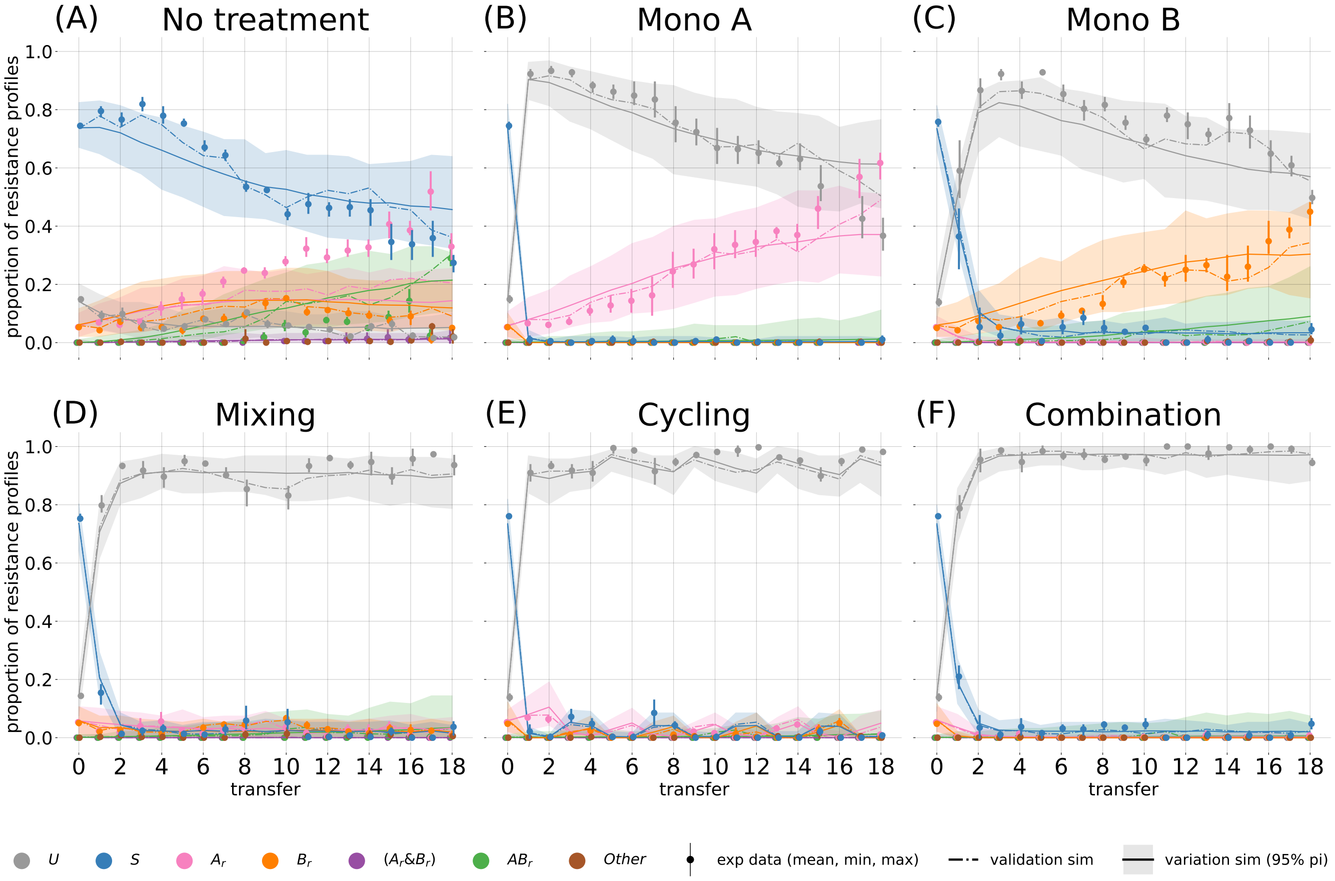

Fig. S2. ***Prevention* scenario:** Frequencies of resistance profiles (colours) over time during the *prevention* scenario. The dots show the experimental measurements, and the error bar indicates the min/max interval between the replicates. The dash-dotted line shows the mean value of 100 stochastic simulations based on the instruction set used in the in vitro experiment. The solid line represents the mean value of 100 simulations with randomly created instruction sets based on the parameter set used in the experiment. The shaded error band indicates the 95-percentile interval between the simulations.

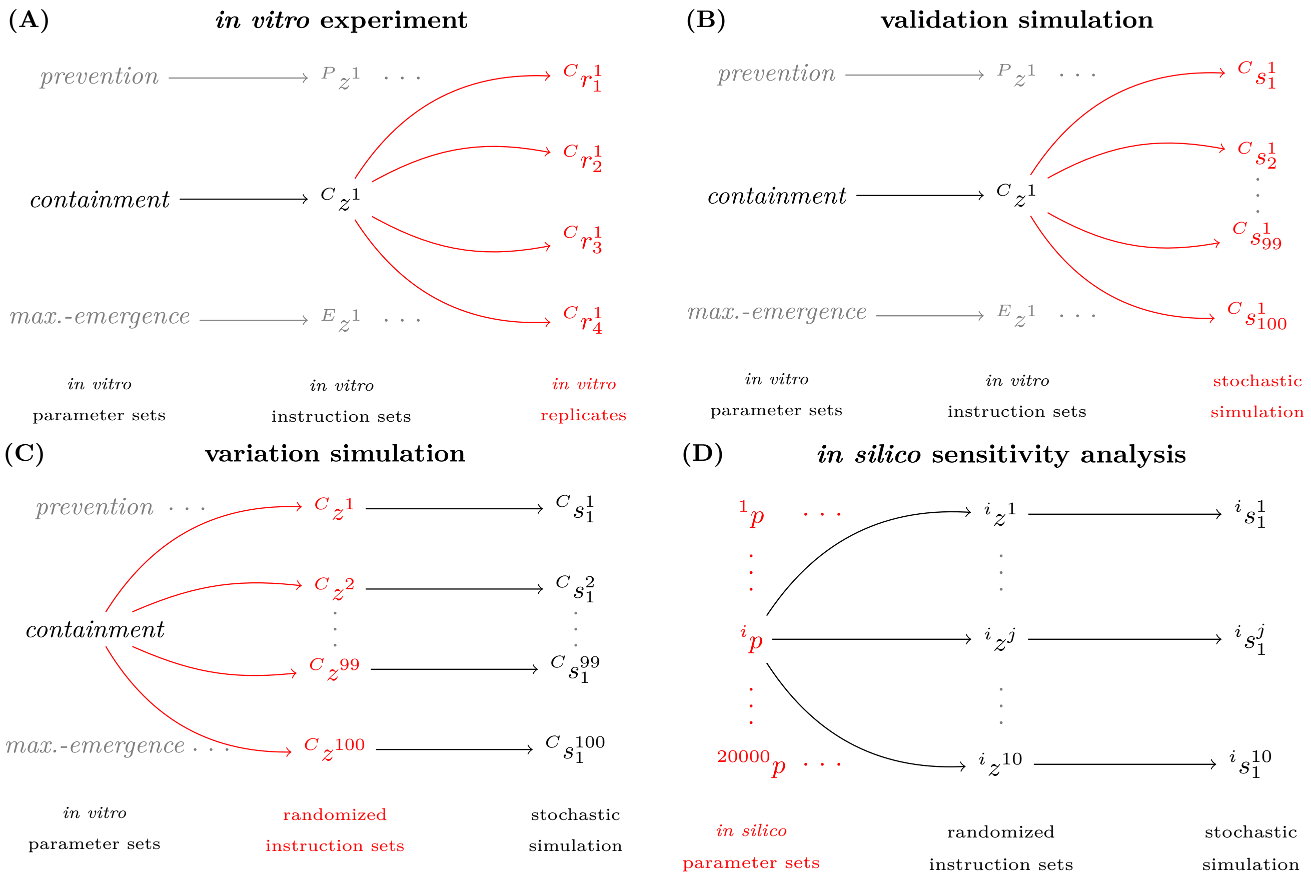

Fig. S3. Illustration depicting the various sources of stochasticity and variation in experiments and simulations. Our experiments and simulations investigate various sources of stochasticity and their contributions to the variability of the outcome. In panels **A**-**D**, we sketch the different sources of stochasticity for each experiment and simulation, highlighting our primary focus in red. **(A)** ***In vitro*** **Experiment.** Each experiment explores a scenario and is defined by a distinct parameter set (*prevention* (P), *containment* (C), *maximum emergence* (E)). We randomly generated one instruction set for each parameter set i: ${}^{i}z^{1}$. For each instruction set ${}^{i}z^{1}$, we replicated the cumulative in-well dynamics four times ${}^{i}r^{1}_{j}$. **(B) Validation Simulation.** To assess our computational model, we employed identical parameter sets and instruction sets ${}^{i}z^{1}$, as employed in the *in vitro* experiments. For each instruction set ${}^{i}z^{1}$, we conducted 100 stochastic simulations ${}^{i}s^{1}_{j}$. **(C) Variation Simulation.** For every *in vitro* parameter set, we randomly generated 100 alternative instruction sets ${}^{i}z^{k}$ to quantify the influence of experimental decisions on the experiment's outcomes. For each instruction set, we performed one simulation ${}^{i}s^{k}_{1}$. **(D)** ***In Silico*** **Sensitivity Analysis.** We examined the sensitivity of our experimental findings to the input parameters by examining the effects of varying the input parameters on the resulting frequency of uninfected cases for different treatment strategies. To achieve this, we generated 20,000 alternative parameter sets ${}^{i}p$. We created ten randomised instruction sets ${}^{i}z^{k}$ for each parameter set ${}^{i}p$ and simulated each instruction set one time (${}^{i}s^{k}_{1}$).

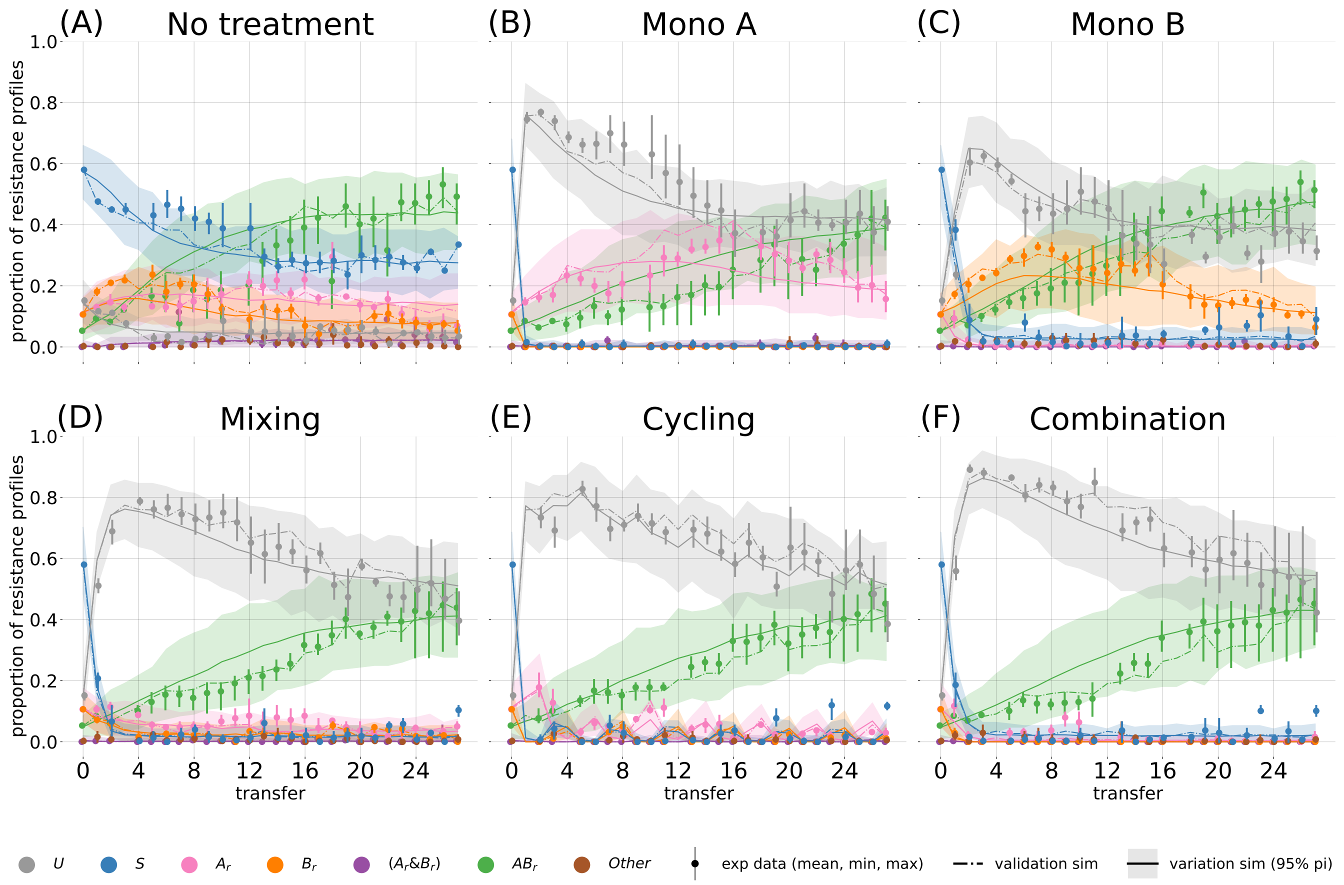

Fig. S4. ***Containment*** **scenario:** Frequencies of resistance profiles (colours) over time during the *containment* scenario. The dots show the experimental measurements, and the error bar indicates the min/max interval between the replicates. The dash-dotted line shows the mean value of 100 stochastic simulations based on the instruction set used in the in vitro experiment. The solid line represents the mean value of 100 simulations with randomly created instruction sets based on the parameter set used in the experiment. The shaded error band indicates the 95-percentile interval between the simulations.

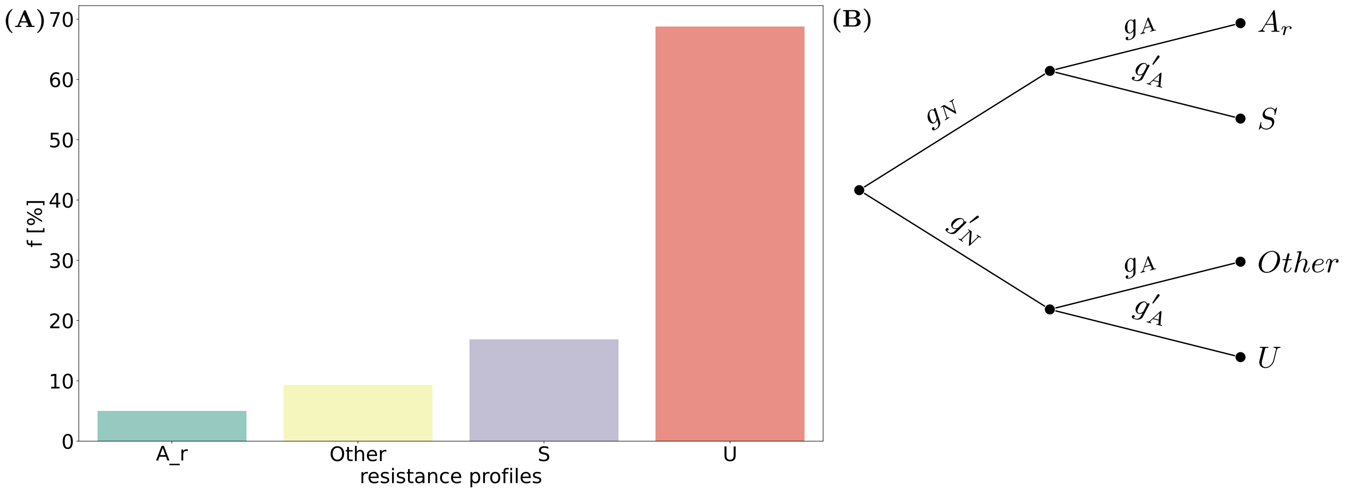

Fig. S5. (A) Experimentally measured resistance profiles for 1784 wells with the pre-treatment profile A_r and treatment with drug B during the *maximum-emergence* scenario. **(B)** Decision tree to calculate the distribution of measured phenotypes for a well that contains $Z_\emptyset$ sensitive and Z_A A-resistant bacteria. g_ϑ is the probability of drawing a drop that forms a colony on a plate treated with drug ϑ, while g'_ϑ is the probability that it does not form a colony.

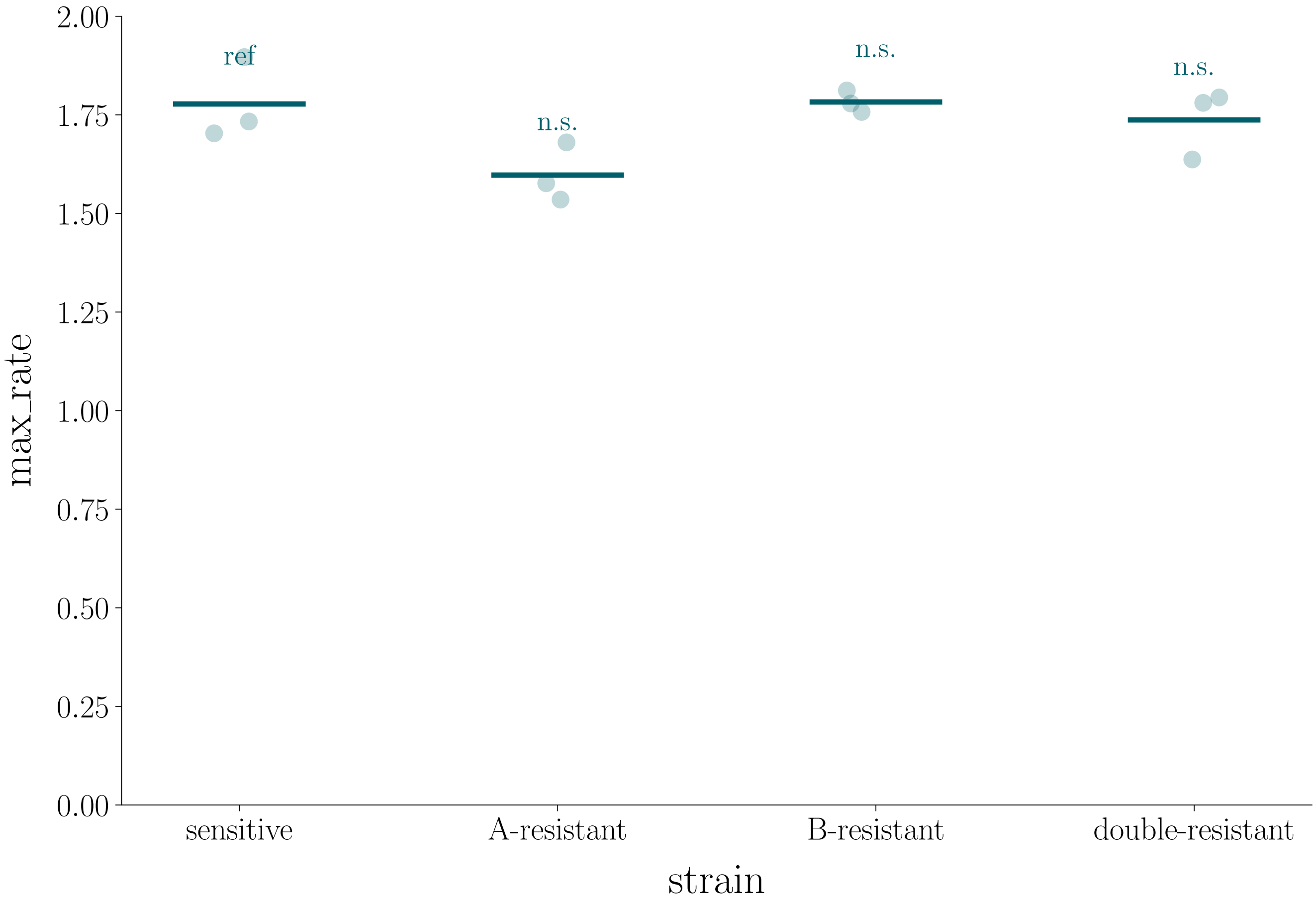

Fig. S6. Maximum growth rates of sensitive and plasmid-carrying strains, measured using OD-growth curves. Each dot represents an individual well, and vertical bars indicate the mean. The sensitive strain was used as the reference ("ref") for pairwise comparisons to the plasmid-carrying strains to identify potential plasmid costs. We used the Mann-Whitney U tests with the Bonferroni correction to identify significant differences in growth rates. All pairwise comparisons were not significant (n.s.).

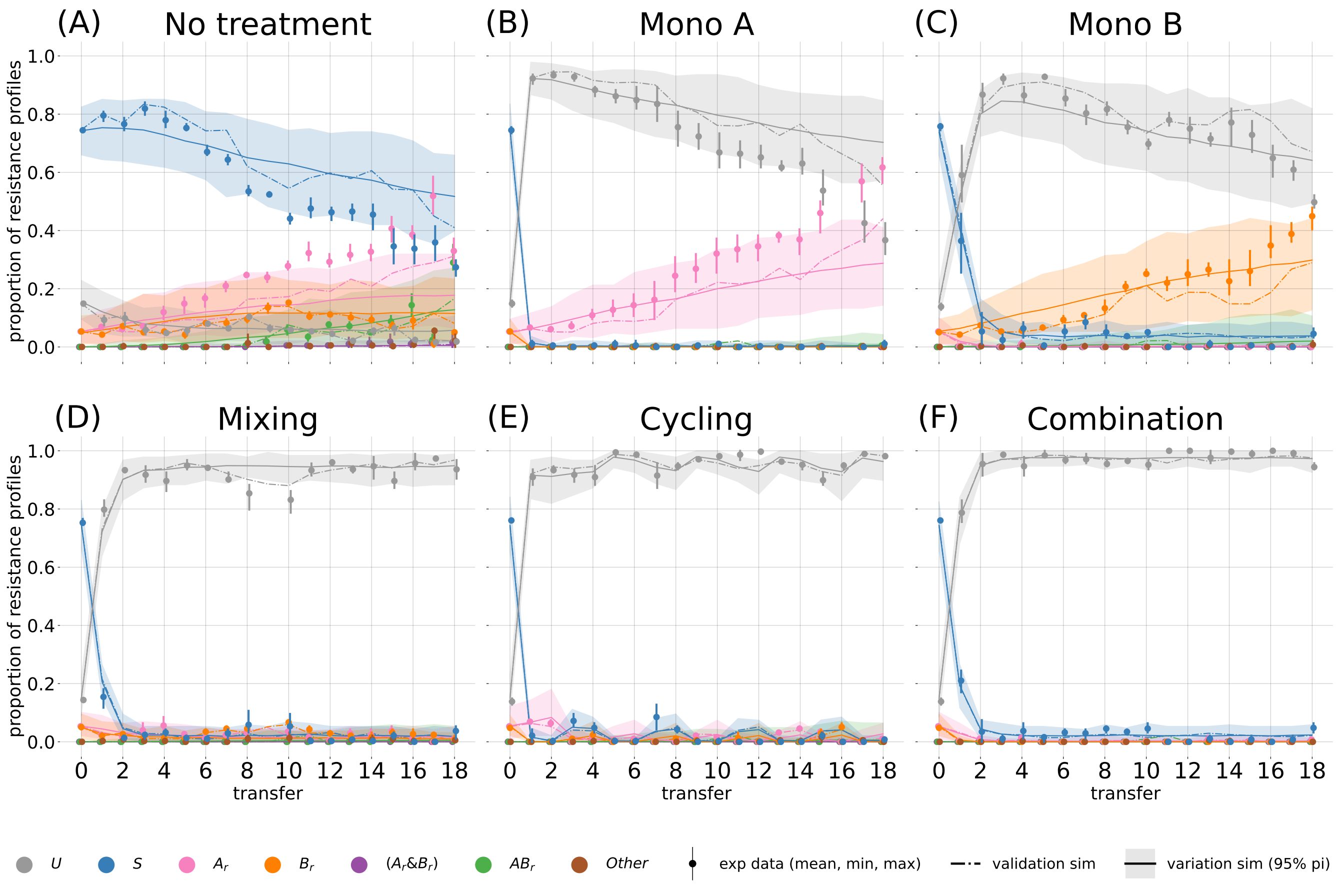

Fig. S7. ***Prevention*** **scenario with filtered transition probabilities.** Frequencies of resistance profiles (colours) over time during the *prevention* scenario. The dots show the experimental measurements, and the error bar indicates the min/max interval between the replicates. The dash-dotted line shows the mean value of 100 stochastic simulations based on the instruction set used in the in vitro experiment. The solid line represents the mean value of 100 simulations with randomly created instruction sets based on the parameter set used in the experiment. The shaded error band indicates the 95-percentile interval between the simulations.

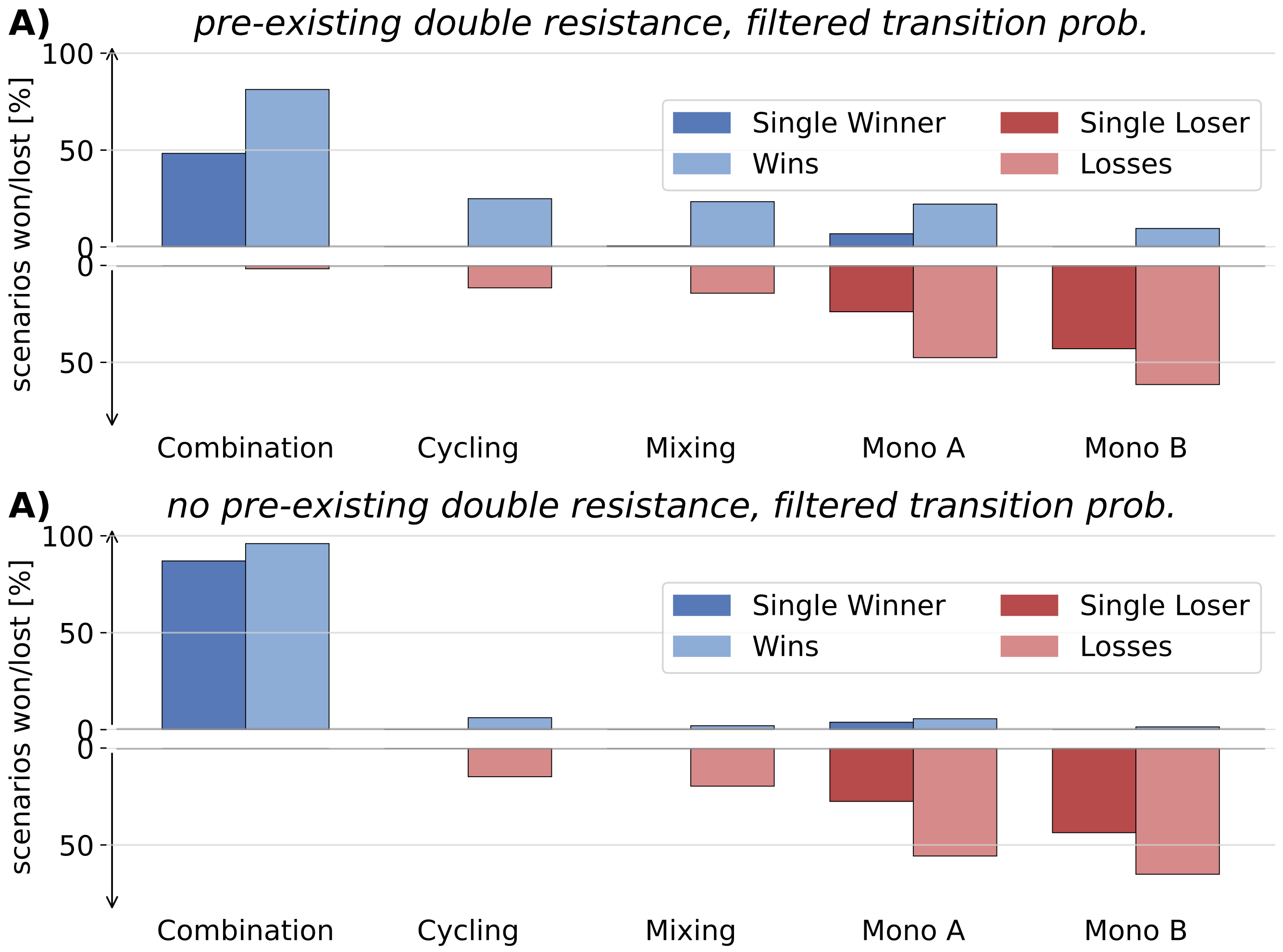

Fig. S8. Sensitivity analysis using filtered transition probabilities. We evaluated the effectiveness of the five treatment strategies in maximising the frequency of uninfected *in silico* patients across randomly generated parameter sets. Strategies not significantly better than any other are marked as losers (pastel red), with those significantly worse than all others being labelled as single losers (dark red). Conversely, strategies that are not significantly worse than any other are classified as winners (pastel blue), and those significantly better than all others as single winners (dark blue). **(A)** Evaluation of 10,000 parameter sets with preexisting double resistance. 659 out of 10,000 parameter sets yielded no significant difference between the strategies. **(B)** Evaluation of 10,000 parameter sets without preexisting double resistance. 8 out of 10,000 parameter sets yielded no significant difference between the strategies.

Table S1. Number of superinfections ($N_{\mathcal{S}}$) between high- and low-concentrated A_r and B_r wells and the number of double resistances that emerged ($N_{\mathcal{E}}$) under treatment ϑ across all three experiments.

Treatment ϑ	$\mathbf{A_r^x}$	$\mathbf{B_r^x}$	$\mathbf{N_{\mathcal{E}}^{\vartheta}}$	$\mathbf{N_{\mathcal{S}}^{\vartheta}}$	$\frac{\mathbf{N_{\mathcal{E}}^{\vartheta}}}{\mathbf{N_{\mathcal{S}}^{\vartheta}}}$
A	A_r^h	B_r^h	55	399	0.14
AB	A_r^h	B_r^h	2	35	0.06
B	A_r^h	B_r^h	257	322	0.8
none	A_r^h	B_r^h	390	420	0.93
A	A_r^h	B_r^l	0	1	0.0
A	A_r^l	B_r^h	0	13	0.0
AB	A_r^l	B_r^h	0	15	0.0
B	A_r^l	B_r^h	0	15	0.0

Table S2. Strains used in this study and their relevant phenotypes. The phenotype in brackets is conferred by the respective plasmid. Cm^R: Chloramphenicol resistance, Amp^R: Ampicillin resistance, Caz^R: Ceftazidime resistance, Tet^R: Tetracycline resistance.

Name	Relevant phenotype	Reference
Escherichia coli MDS42-YFP	Cm^R	(3)
A-resistant	Cm^R pA (Amp^R, Caz^R)	this study, (2)
B-resistant	Cm^R pB (Amp^R, Tet^R)	this study, (2)
AB-resistant	Cm^R pA (Amp^R, Caz^R) pB (Amp^R, Tet^R)	this study, (2)

Table S3. Statistical comparison of maximum growth rates between the sensitive and plasmid-carrying strains. We used a Mann-Whitney U test for pairwise comparisons, and the p-values were adjusted using the Bonferroni correction.

Comparison	U-statistic	P-value	Corrected P-value	Significant after Bonferroni
sensitive vs A-resistant	9.000000	0.100000	0.300000	False
sensitive vs B-resistant	3.000000	0.700000	1.000000	False
sensitive vs double-resistant	5.000000	1.000000	1.000000	False

Table S4. Plasmid segregation loss was estimated over 24 hours without treatment and with selective treatment as a control. Frequencies of plasmid retention were compared between t_0 – t_1 using the Mann-Whitney U test. Confidence intervals and the mean frequencies were estimated by bootstrapping the binary data (plasmid retained or lost) pooled across replicates. No significant plasmid loss was observed in either the main data or the control.

plasmids	$f(t_0)$	$CI(t_0)$	$f(t_1)$	$CI(t_1)$	p-value	$f(t_1)$ control	$CI(t_1)$ control	p-value control
p_A	1.00	(1.0, 1.0)	1.00	(1.0, 1.0)	1.00	1.00	(1.0, 1.0)	1.00
p_B	1.00	(1.0, 1.0)	0.97	(0.93, 1.0)	0.20	1.00	(1.0, 1.0)	1.00
p_A&p_B	0.99	(0.97, 1.0)	1.00	(1.0, 1.0)	0.50	1.00	(1.0, 1.0)	0.50

Table S5. 95 % confidence intervals for the final bacterial density measured by colony plating and the maximal growth rates measured by evaluating OD-growth curves.

strain	antibiotic	cmp $[\mu g/ml]$	cfu $[1/\mu l]$	growthrate $[1/h]$
double-resistant	AB	5	$(2.14 - 3.33) \times 10^5$	$(0.43 - 0.86)$
double-resistant	AB	25	$(2.68 - 3.99) \times 10^5$	$(0.4 - 0.85)$
A-resistant	A	5	$(1.58 - 2.62) \times 10^5$	$(0.51 - 0.7)$
A-resistant	A	25	$(2.05 - 3.21) \times 10^5$	$(0.46 - 0.71)$
sensitive	None	5	$(1.03 - 1.27) \times 10^6$	$(0.68 - 0.74)$
sensitive	None	25	$(1.15 - 1.4) \times 10^6$	$(0.55 - 0.96)$
B-resistant	B	5	$(4.05 - 5.62) \times 10^5$	$(0.45 - 0.74)$
B-resistant	B	25	$(3.53 - 5.01) \times 10^5$	$(0.4 - 0.87)$

Table S6. Definition of resistance profiles (rows) by growth patterns on differently treated *agar plates* (columns). X indicates colony formation, whereas o indicates no growth.

	None	A	B	AB
U	o	o	o	o
S	X	o	o	o
A_r	X	X	o	o
B_r	X	o	X	o
$(A_r\&B_r)$	X	X	X	o
AB_r	X	X	X	X

Table S7. Association between bacterial phenotypes (rows) and resistance profiles ϕ (columns). An 'X' denotes that a phenotype is obligatory for a given profile, while a '$\surd$' indicates that it is optional.

	U	S	A_r	B_r	$(A_r\&B_r)$	AB_r
sensitive		X	$\surd$	$\surd$	$\surd$	$\surd$
A-resistant			X		X	$\surd$
B-resistant				X	X	$\surd$
double-resistant						X

Table S8. Clearance probability of well phenotypes across the three experiments.

	S	A_r	B_r
A	0.97	0.02	0.98
B	0.73	0.62	0.01
AB	0.86	0.4	0.98

Table S9. Mixing rules. During the plate-handling phase, we mix wells due to infections. The resulting phenotype of the two mixed wells can be calculated using this table. More than two phenotypes can be combined by applying associative logic.

$\phi_1(\hat{T})$ \ $\phi_2(\hat{T})$	U	S	A_r	B_r	$(A_r\&B_r)$	AB_r
U	U	S	A_r	B_r	$(A_r\&B_r)$	AB_r
S	S	S	A_r	B_r	$(A_r\&B_r)$	AB_r
A_r	A_r	A_r	A_r	$(A_r\&B_r)$	$(A_r\&B_r)$	AB_r
B_r	B_r	B_r	$(A_r\&B_r)$	B_r	$(A_r\&B_r)$	AB_r
(A_r&B_r)	$(A_r\&B_r)$	$(A_r\&B_r)$	$(A_r\&B_r)$	$(A_r\&B_r)$	$(A_r\&B_r)$	AB_r
AB_r	AB_r	AB_r	AB_r	AB_r	AB_r	AB_r

Table S10. Mean parameter leading to n single wins during the sensitivity analysis without preexisting double resistance. Strategies that did not yield at least one single win were excluded.

	turnover	infection	U	S	A_r	B_r	AB_r	n
Combination	0.50	0.50	0.26	0.24	0.24	0.25	0.00	9311.00
Mono A	0.80	0.27	0.26	0.29	0.37	0.08	0.00	56.00
Cycling	0.10	0.39	0.33	0.42	0.14	0.10	0.00	4.00
Mixing	0.11	0.33	0.12	0.49	0.38	0.01	0.00	1.00

Table S11. Mean parameter leading to n single losses during the sensitivity analysis without preexisting double resistance. Strategies that did not yield at least one single loss were excluded.

	turnover	infection	U	S	A_r	B_r	AB_r	n
Mono B	0.60	0.43	0.26	0.25	0.16	0.33	0.00	4132.00
Mono A	0.55	0.43	0.27	0.25	0.36	0.12	0.00	2648.00
Cycling	0.07	0.87	0.28	0.34	0.27	0.11	0.00	8.00
Mixing	0.07	0.90	0.69	0.25	0.01	0.05	0.00	1.00

Table S12. Mean parameter leading to n single wins during the sensitivity analysis with preexisting double resistance. Strategies that did not yield at least one single win were excluded.

	turnover	infection	U	S	A_r	B_r	AB_r	n
Combination	0.61	0.47	0.21	0.18	0.22	0.22	0.17	5487.00
Mono A	0.52	0.56	0.23	0.20	0.16	0.15	0.26	365.00
Mixing	0.13	0.30	0.23	0.21	0.18	0.15	0.22	40.00
Cycling	0.14	0.20	0.23	0.20	0.21	0.20	0.16	9.00

Table S13. Mean parameter leading to n single losses during the sensitivity analysis with preexisting double resistance. Strategies that did not yield at least one single loss were excluded.

	turnover	infection	U	S	A_r	B_r	AB_r	n
Mono B	0.57	0.46	0.21	0.19	0.13	0.28	0.18	4250.00
Mono A	0.51	0.43	0.23	0.19	0.30	0.10	0.18	2359.00
Cycling	0.42	0.72	0.23	0.21	0.14	0.14	0.28	6.00
Mixing	0.34	0.71	0.23	0.20	0.18	0.09	0.30	2.00

Table S14. Wins and losses during the sensitivity analysis. With preexisting double resistance. 606 parameter sets yielded an insignificant result.

strategy	single winner [%]	single loser [%]	loser [%]	winner [%]	single winner	single loser	loser	winner
Combination	54.87	0.00	0.95	86.76	5487	0	95	8676
Cycling	0.09	0.06	12.10	23.32	9	6	1210	2332
Mixing	0.40	0.02	14.78	20.54	40	2	1478	2054
Mono A	3.65	23.59	48.57	17.49	365	2359	4857	1749
Mono B	0.00	42.50	62.29	8.11	0	4250	6229	811

Table S15. Wins and losses during the sensitivity analysis without preexisting double resistance. 100 parameter sets yielded an insignificant result.

strategy	single winner [%]	single loser [%]	loser [%]	winner [%]	single winner	single loser	loser	winner
Combination	93.11	0.00	0.00	98.35	9311	0	0	9835
Cycling	0.04	0.08	18.89	3.49	4	8	1889	349
Mixing	0.01	0.01	22.05	1.32	1	1	2205	132
Mono A	0.56	26.48	57.03	1.65	56	2648	5703	165
Mono B	0.00	41.32	63.44	1.11	0	4132	6344	111

Table S16. Wins and losses during the sensitivity analysis, using filtered transition probabilities and preexisting double resistance. 659 parameter sets yielded an insignificant result.

strategy	single winner [%]	single loser [%]	loser [%]	winner [%]	single winner	single loser	loser	winner
Combination	48.31	0.00	1.72	81.27	4831	0	172	8127
Cycling	0.07	0.10	11.57	24.94	7	10	1157	2494
Mixing	0.59	0.03	14.34	23.40	59	3	1434	2340
Mono A	6.85	23.91	47.57	22.15	685	2391	4757	2215
Mono B	0.00	42.97	61.45	9.57	0	4297	6145	957

Table S17. Wins and losses during the sensitivity analysis with filtered transition matrices and no preexisting double resistance. 8 parameter sets yielded an insignificant result.

strategy	single winner [%]	single loser [%]	loser [%]	winner [%]	single winner	single loser	loser	winner
Combination	87.04	0.00	0.00	95.98	8704	0	0	9598
Cycling	0.08	0.06	14.80	6.16	8	6	1480	616
Mixing	0.00	0.04	19.66	2.00	0	4	1966	200
Mono A	3.82	27.53	55.71	5.62	382	2753	5571	562
Mono B	0.00	43.67	65.10	1.41	0	4367	6510	141

Table S18. M^{none}**. Unfiltered transition matrix for the untreated wells.**

$\phi(\hat{T})$ \ $\phi(T)$	U	S	A_r	B_r	$(A_r\&B_r)$	AB_r
U	0.97	0.0	0.0	0.0	0.0	0.0
S	0.03	0.98	0.01	0.02	0.01	0.01
A_r	0.0	0.01	0.96	0.0	0.07	0.03
B_r	0.0	0.01	0.0	0.97	0.02	0.0
(A_r&B_r)	0.0	0.0	0.01	0.01	0.08	0.04
AB_r	0.0	0.0	0.02	0.0	0.82	0.92

Table S19. M^{A}**. Unfiltered transition matrix for wells treated with antibiotic A.**

$\phi(\hat{T})$ \ $\phi(T)$	U	S	A_r	B_r	$(A_r\&B_r)$	AB_r
U	0.99	0.96	0.02	0.97	0.03	0.02
S	0.0	0.02	0.0	0.01	0.0	0.0
A_r	0.01	0.02	0.98	0.01	0.59	0.01
B_r	0.0	0.0	0.0	0.01	0.0	0.0
(A_r&B_r)	0.0	0.0	0.0	0.0	0.18	0.01
AB_r	0.0	0.0	0.0	0.0	0.2	0.96

Table S20. M^{B}**. Unfiltered transition matrix for wells treated with antibiotic B.**

$\phi(\hat{T})$ \ $\phi(T)$	U	S	A_r	B_r	$(A_r\&B_r)$	AB_r
U	0.99	0.8	0.63	0.01	0.03	0.02
S	0.0	0.19	0.18	0.0	0.0	0.0
A_r	0.0	0.0	0.18	0.0	0.0	0.0
B_r	0.01	0.01	0.0	0.98	0.21	0.0
(A_r&B_r)	0.0	0.0	0.0	0.0	0.03	0.0
AB_r	0.0	0.0	0.01	0.01	0.73	0.98

Table S21. M^{AB}**. Unfiltered transition matrix for wells treated with antibiotic AB.**

$\phi(\hat{T})$ \ $\phi(T)$	U	S	A_r	B_r	$(A_r\&B_r)$	AB_r
U	1.0	0.9	0.49	0.98	1.0	0.02
S	0.0	0.1	0.32	0.02	0.0	0.0
A_r	0.0	0.0	0.19	0.0	0.0	0.0
B_r	0.0	0.0	0.0	0.0	0.0	0.0
(A_r&B_r)	0.0	0.0	0.0	0.0	0.0	0.0
AB_r	0.0	0.0	0.0	0.0	0.0	0.98

Table S22. M_1^{none}**. Unfiltered transition matrix for the first time point in untreated wells.**

$\phi(\hat{T})$ \ $\phi(T)$	U	S	A_r	B_r	$(A_r\&B_r)$	AB_r
U	0.96	0.0	0.0	0.0	0.0	0.0
S	0.03	0.99	0.0	0.0	0.0	0.0
A_r	0.01	0.01	1.0	0.0	0.0	0.0
B_r	0.0	0.0	0.0	1.0	0.17	0.0
(A_r&B_r)	0.0	0.0	0.0	0.0	0.02	0.0
AB_r	0.0	0.0	0.0	0.0	0.81	1.0

Table S23. M_1^{A}**. Unfiltered transition matrix for the first timepoint in wells treated with antibiotic A.**

$\phi(\hat{T})$ \ $\phi(T)$	U	S	A_r	B_r	$(A_r\&B_r)$	AB_r
U	0.99	0.97	0.0	0.99	0.23	0.0
S	0.0	0.02	0.0	0.01	0.0	0.0
A_r	0.01	0.01	1.0	0.0	0.54	0.0
B_r	0.0	0.0	0.0	0.0	0.0	0.0
(A_r&B_r)	0.0	0.0	0.0	0.0	0.11	0.0
AB_r	0.0	0.0	0.0	0.0	0.12	1.0

Table S24. M_1^{B}**. Unfiltered transition matrix for the first time point in wells treated with antibiotic B.**

$\phi(\hat{T})$ \ $\phi(T)$	U	S	A_r	B_r	$(A_r\&B_r)$	AB_r
U	1.0	0.48	0.51	0.02	0.01	0.0
S	0.0	0.52	0.21	0.01	0.0	0.0
A_r	0.0	0.0	0.28	0.0	0.0	0.0
B_r	0.0	0.0	0.0	0.97	0.31	0.0
(A_r&B_r)	0.0	0.0	0.0	0.0	0.1	0.0
AB_r	0.0	0.0	0.0	0.0	0.58	1.0

Table S25. M_1^{AB}**. Unfiltered transition matrix for the first time point in wells treated with antibiotic AB.**

$\phi(\hat{T})$ \ $\phi(T)$	U	S	A_r	B_r	$(A_r\&B_r)$	AB_r
U	0.98	0.79	0.32	0.67	0.24	0.0
S	0.02	0.21	0.21	0.24	0.21	0.0
A_r	0.0	0.0	0.47	0.0	0.41	0.0
B_r	0.0	0.0	0.0	0.09	0.02	0.0
(A_r&B_r)	0.0	0.0	0.0	0.0	0.07	0.0
AB_r	0.0	0.0	0.0	0.0	0.05	1.0

Table S26. M^{none}. **Filtered transition matrix for untreated wells.**

$\phi(\hat{T})$ \ $\phi(T)$	U	S	A_r	B_r	$(A_r\&B_r)$	AB_r
U	1.0	0.0	0.0	0.0	0.0	0.0
S	0.0	1.0	0.01	0.02	0.01	0.01
A_r	0.0	0.0	0.99	0.0	0.07	0.03
B_r	0.0	0.0	0.0	0.98	0.02	0.0
(A_r&B_r)	0.0	0.0	0.0	0.0	0.08	0.04
AB_r	0.0	0.0	0.0	0.0	0.82	0.92

Table S27. M^{A}. **Filtered transition matrix for wells treated with antibiotic A.**

$\phi(\hat{T})$ \ $\phi(T)$	U	S	A_r	B_r	$(A_r\&B_r)$	AB_r
U	1.0	0.98	0.02	0.98	0.03	0.02
S	0.0	0.02	0.0	0.01	0.0	0.0
A_r	0.0	0.0	0.98	0.0	0.59	0.01
B_r	0.0	0.0	0.0	0.01	0.0	0.0
(A_r&B_r)	0.0	0.0	0.0	0.0	0.18	0.01
AB_r	0.0	0.0	0.0	0.0	0.2	0.96

Table S28. M^{B}. **Filtered transition matrix for wells treated with antibiotic B.**

$\phi(\hat{T})$ \ $\phi(T)$	U	S	A_r	B_r	$(A_r\&B_r)$	AB_r
U	1.0	0.8	0.64	0.01	0.03	0.02
S	0.0	0.2	0.18	0.0	0.0	0.0
A_r	0.0	0.0	0.18	0.0	0.0	0.0
B_r	0.0	0.0	0.0	0.99	0.21	0.0
(A_r&B_r)	0.0	0.0	0.0	0.0	0.03	0.0
AB_r	0.0	0.0	0.0	0.0	0.73	0.98

Table S29. M^{AB}. **Filtered transition matrix for wells treated with antibiotic AB.**

$\phi(\hat{T})$ \ $\phi(T)$	U	S	A_r	B_r	$(A_r\&B_r)$	AB_r
U	1.0	0.9	0.49	0.98	1.0	0.02
S	0.0	0.1	0.32	0.02	0.0	0.0
A_r	0.0	0.0	0.19	0.0	0.0	0.0
B_r	0.0	0.0	0.0	0.0	0.0	0.0
(A_r&B_r)	0.0	0.0	0.0	0.0	0.0	0.0
AB_r	0.0	0.0	0.0	0.0	0.0	0.98

Table S30. M_1^{none}. **Filtered transition matrix for the first time point in untreated wells.**

$\phi(\hat{T})$ \ $\phi(T)$	U	S	A_r	B_r	$(A_r\&B_r)$	AB_r
U	1.0	0.0	0.0	0.0	0.0	0.0
S	0.0	1.0	0.0	0.0	0.0	0.0
A_r	0.0	0.0	1.0	0.0	0.0	0.0
B_r	0.0	0.0	0.0	1.0	0.17	0.0
(A_r&B_r)	0.0	0.0	0.0	0.0	0.02	0.0
AB_r	0.0	0.0	0.0	0.0	0.81	1.0

Table S31. M_1^{A}. **Filtered transition matrix for the first time point in wells treated with antibiotic A.**

$\phi(\hat{T})$ \ $\phi(T)$	U	S	A_r	B_r	$(A_r\&B_r)$	AB_r
U	1.0	0.98	0.0	0.99	0.23	0.0
S	0.0	0.02	0.0	0.01	0.0	0.0
A_r	0.0	0.0	1.0	0.0	0.54	0.0
B_r	0.0	0.0	0.0	0.0	0.0	0.0
(A_r&B_r)	0.0	0.0	0.0	0.0	0.11	0.0
AB_r	0.0	0.0	0.0	0.0	0.12	1.0

Table S32. M_1^{B}. **Filtered transition matrix for the first time point in wells treated with antibiotic B.**

$\phi(\hat{T})$ \ $\phi(T)$	U	S	A_r	B_r	$(A_r\&B_r)$	AB_r
U	1.0	0.48	0.51	0.02	0.01	0.0
S	0.0	0.52	0.21	0.01	0.0	0.0
A_r	0.0	0.0	0.28	0.0	0.0	0.0
B_r	0.0	0.0	0.0	0.97	0.31	0.0
(A_r&B_r)	0.0	0.0	0.0	0.0	0.1	0.0
AB_r	0.0	0.0	0.0	0.0	0.58	1.0

Table S33. M_1^{AB}. **Filtered transition matrix for the first time point in wells treated with antibiotic AB.**

$\phi(\hat{T})$ \ $\phi(T)$	U	S	A_r	B_r	$(A_r\&B_r)$	AB_r
U	1.0	0.79	0.32	0.67	0.24	0.0
S	0.0	0.21	0.21	0.24	0.21	0.0
A_r	0.0	0.0	0.47	0.0	0.41	0.0
B_r	0.0	0.0	0.0	0.09	0.02	0.0
(A_r&B_r)	0.0	0.0	0.0	0.0	0.07	0.0
AB_r	0.0	0.0	0.0	0.0	0.05	1.0

Table S34. ***Prevention*** **scenario: Effect of treatment strategies on the frequency of uninfecteds (ANOVA).**

	Sum of Squares	df	Mean Square	F	Sig.
Between Groups	2.833	5	0.567	779.436	< 0.001
Within Groups	0.013	18	< 0.001		
Total	2.846	23			

Table S35. ***Prevention*** **scenario: Multiple comparison between the effects of treatment strategies on the frequencies of uninfecteds using Tukey's post-hoc analysis.**

group1	group2	meandiff	p-adj	lower	upper	reject
Combination	Cycling	-0.027	0.729	-0.087	0.034	False
Combination	Mixing	-0.041	0.317	-0.101	0.020	False
Combination	Mono A	-0.525	0.000	-0.586	-0.465	True
Combination	Mono B	-0.360	0.000	-0.421	-0.300	True
Combination	No treatment	-0.951	0.000	-1.011	-0.890	True
Cycling	Mixing	-0.014	0.975	-0.074	0.047	False
Cycling	Mono A	-0.499	0.000	-0.559	-0.438	True
Cycling	Mono B	-0.334	0.000	-0.394	-0.273	True
Cycling	No treatment	-0.924	0.000	-0.985	-0.864	True
Mixing	Mono A	-0.485	0.000	-0.545	-0.424	True
Mixing	Mono B	-0.320	0.000	-0.380	-0.259	True
Mixing	No treatment	-0.910	0.000	-0.971	-0.850	True
Mono A	Mono B	0.165	0.000	0.104	0.226	True
Mono A	No treatment	-0.425	0.000	-0.486	-0.365	True
Mono B	No treatment	-0.590	0.000	-0.651	-0.530	True

Table S36. ***Prevention*** **scenario: Effect of treatment strategies on the frequency of single resistance (ANOVA).**

	Sum of Squares	df	Mean Square	F	Sig.
Between Groups	1.133	5	0.227	290.494	< 0.001
Within Groups	0.014	18	< 0.001		
Total	1.147	23			

Table S37. ***Prevention*** **scenario: Multiple comparison between the effects of treatment strategies on the frequencies of single resistance using Tukey's post-hoc analysis.**

group1	group2	meandiff	p-adj	lower	upper	reject
Combination	Cycling	0.030	0.659	-0.033	0.093	False
Combination	Mixing	0.035	0.518	-0.028	0.097	False
Combination	Mono A	0.501	0.000	0.438	0.563	True
Combination	Mono B	0.359	0.000	0.296	0.422	True
Combination	No treatment	0.479	0.000	0.417	0.542	True
Cycling	Mixing	0.005	1.000	-0.058	0.067	False
Cycling	Mono A	0.471	0.000	0.408	0.533	True
Cycling	Mono B	0.329	0.000	0.266	0.392	True
Cycling	No treatment	0.450	0.000	0.387	0.512	True
Mixing	Mono A	0.466	0.000	0.403	0.529	True
Mixing	Mono B	0.325	0.000	0.262	0.387	True
Mixing	No treatment	0.445	0.000	0.382	0.508	True
Mono A	Mono B	-0.142	0.000	-0.204	-0.079	True
Mono A	No treatment	-0.021	0.884	-0.084	0.042	False
Mono B	No treatment	0.120	0.000	0.058	0.183	True

Table S38. ***Prevention*** **scenario: Effect of treatment strategies on the frequency of double resistance (ANOVA).**

	Sum of Squares	df	Mean Square	F	Sig.
Between Groups	0.061	5	0.012	157.486	< 0.001
Within Groups	0.001	18	< 0.001		
Total	0.063	23			

Table S39. ***Prevention*** **scenario: Effect of treatment strategies on the frequency of double resistance (ANOVA).**

group1	group2	meandiff	p-adj	lower	upper	reject
Combination	Cycling	0.000	1.000	-0.020	0.020	False
Combination	Mixing	0.003	0.998	-0.017	0.022	False
Combination	Mono A	0.002	1.000	-0.018	0.022	False
Combination	Mono B	0.000	1.000	-0.020	0.020	False
Combination	No treatment	0.136	0.000	0.117	0.156	True
Cycling	Mixing	0.003	0.998	-0.017	0.022	False
Cycling	Mono A	0.002	1.000	-0.018	0.022	False
Cycling	Mono B	0.000	1.000	-0.020	0.020	False
Cycling	No treatment	0.136	0.000	0.117	0.156	True
Mixing	Mono A	-0.001	1.000	-0.021	0.019	False
Mixing	Mono B	-0.003	0.998	-0.022	0.017	False
Mixing	No treatment	0.134	0.000	0.114	0.153	True
Mono A	Mono B	-0.002	1.000	-0.022	0.018	False
Mono A	No treatment	0.134	0.000	0.115	0.154	True
Mono B	No treatment	0.136	0.000	0.117	0.156	True

Table S40. ***Containment*** **scenario: Effect of treatment strategies on the frequency of uninfecteds (ANOVA).**

	Sum of Squares	df	Mean Square	F	Sig.
Between Groups	0.639	5	0.128	28.906	< 0.001
Within Groups	0.080	18	0.004		
Total	0.718	23			

Table S41. ***Containment*** **scenario: Multiple comparison between the effects of treatment strategies on the frequencies of uninfecteds using Tukey's post-hoc analysis.**

group1	group2	meandiff	p-adj	lower	upper	reject
Combination	Cycling	-0.008	1.000	-0.157	0.141	False
Combination	Mixing	-0.041	0.951	-0.190	0.109	False
Combination	Mono A	-0.104	0.282	-0.253	0.046	False
Combination	Mono B	-0.158	0.034	-0.308	-0.009	True
Combination	No treatment	-0.474	0.000	-0.623	-0.325	True
Cycling	Mixing	-0.033	0.980	-0.182	0.117	False
Cycling	Mono A	-0.096	0.361	-0.245	0.054	False
Cycling	Mono B	-0.150	0.048	-0.300	-0.001	True
Cycling	No treatment	-0.466	0.000	-0.616	-0.317	True
Mixing	Mono A	-0.063	0.758	-0.212	0.086	False
Mixing	Mono B	-0.118	0.175	-0.267	0.032	False
Mixing	No treatment	-0.433	0.000	-0.583	-0.284	True
Mono A	Mono B	-0.054	0.850	-0.204	0.095	False
Mono A	No treatment	-0.370	0.000	-0.520	-0.221	True
Mono B	No treatment	-0.316	0.000	-0.465	-0.166	True

Table S42. ***Containment*** **scenario: Effect of treatment strategies on the frequency of single resistance (ANOVA).**

	Sum of Squares	df	Mean Square	F	Sig.
Between Groups	0.129	5	0.026	40.881	< 0.001
Within Groups	0.011	18	< 0.001		
Total	0.140	23			

Table S43. *Containment* scenario: Multiple comparison between the effects of treatment strategies on the frequencies of single resistance using Tukey's post-hoc analysis.

group1	group2	meandiff	p-adj	lower	upper	reject
Combination	Cycling	0.019	0.895	-0.038	0.075	False
Combination	Mixing	0.054	0.067	-0.003	0.110	False
Combination	Mono A	0.197	0.000	0.140	0.253	True
Combination	Mono B	0.102	0.000	0.045	0.158	True
Combination	No treatment	0.169	0.000	0.112	0.225	True
Cycling	Mixing	0.035	0.389	-0.021	0.092	False
Cycling	Mono A	0.178	0.000	0.122	0.235	True
Cycling	Mono B	0.083	0.002	0.027	0.140	True
Cycling	No treatment	0.150	0.000	0.094	0.207	True
Mixing	Mono A	0.143	0.000	0.086	0.199	True
Mixing	Mono B	0.048	0.125	-0.009	0.104	False
Mixing	No treatment	0.115	0.000	0.059	0.172	True
Mono A	Mono B	-0.095	0.001	-0.152	-0.039	True
Mono A	No treatment	-0.028	0.626	-0.084	0.029	False
Mono B	No treatment	0.067	0.015	0.011	0.124	True

Table S44. *Containment* scenario: Effect of treatment strategies on the frequency of double resistance (ANOVA).

	Sum of Squares	df	Mean Square	F	Sig.
Between Groups	0.038	5	0.008	1.169	0.362
Within Groups	0.116	18	0.006		
Total	0.154	23			

Table S45. *Maximum-emergence* scenario: Effect of treatment strategies on the frequency of uninfecteds (ANOVA).

	Sum of Squares	df	Mean Square	F	Sig.
Between Groups	1.432	5	0.286	383.054	< 0.001
Within Groups	0.013	18	< 0.001		
Total	1.445	23			

Table S46. *Maximum-emergence* scenario: Multiple comparison between the effects of treatment strategies on the frequencies of uninfecteds using Tukey's post-hoc analysis.

group1	group2	meandiff	p-adj	lower	upper	reject
Combination	Cycling	-0.306	0.000	-0.368	-0.245	True
Combination	Mixing	-0.386	0.000	-0.447	-0.324	True
Combination	Mono A	-0.414	0.000	-0.476	-0.353	True
Combination	Mono B	-0.499	0.000	-0.561	-0.438	True
Combination	No treatment	-0.823	0.000	-0.885	-0.762	True
Cycling	Mixing	-0.079	0.008	-0.141	-0.018	True
Cycling	Mono A	-0.108	0.000	-0.169	-0.046	True
Cycling	Mono B	-0.193	0.000	-0.254	-0.131	True
Cycling	No treatment	-0.517	0.000	-0.578	-0.455	True
Mixing	Mono A	-0.029	0.681	-0.090	0.033	False
Mixing	Mono B	-0.114	0.000	-0.175	-0.052	True
Mixing	No treatment	-0.438	0.000	-0.499	-0.376	True
Mono A	Mono B	-0.085	0.004	-0.146	-0.024	True
Mono A	No treatment	-0.409	0.000	-0.470	-0.347	True
Mono B	No treatment	-0.324	0.000	-0.385	-0.262	True

Table S47. *Maximum-emergence* scenario: Effect of treatment strategies on the frequency of single resistance (ANOVA).

	Sum of Squares	df	Mean Square	F	Sig.
Between Groups	1.311	5	0.262	524.241	< 0.001
Within Groups	0.009	18	< 0.001		
Total	1.320	23			

Table S48. *Maximum-emergence* scenario: Multiple comparison between the effects of treatment strategies on the frequencies of single resistance using Tukey's post-hoc analysis.

group1	group2	meandiff	p-adj	lower	upper	reject
Combination	Cycling	0.342	0.000	0.292	0.393	True
Combination	Mixing	0.408	0.000	0.358	0.459	True
Combination	Mono A	0.549	0.000	0.499	0.600	True
Combination	Mono B	0.536	0.000	0.486	0.586	True
Combination	No treatment	0.761	0.000	0.710	0.811	True
Cycling	Mixing	0.066	0.006	0.016	0.116	True
Cycling	Mono A	0.207	0.000	0.157	0.257	True
Cycling	Mono B	0.194	0.000	0.143	0.244	True
Cycling	No treatment	0.418	0.000	0.368	0.469	True
Mixing	Mono A	0.141	0.000	0.091	0.191	True
Mixing	Mono B	0.128	0.000	0.077	0.178	True
Mixing	No treatment	0.352	0.000	0.302	0.403	True
Mono A	Mono B	-0.013	0.956	-0.064	0.037	False
Mono A	No treatment	0.211	0.000	0.161	0.262	True
Mono B	No treatment	0.225	0.000	0.174	0.275	True

Table S49. *Maximum-emergence* scenario: Effect of treatment strategies on the frequency of double resistance (ANOVA).

	Sum of Squares	df	Mean Square	F	Sig.
Between Groups	0.109	5	0.022	71.779	< 0.001
Within Groups	0.005	18	< 0.001		
Total	0.115	23			

Table S50. *Maximum-emergence* scenario: Effect of treatment strategies on the frequency of double resistance (ANOVA).

group1	group2	meandiff	p-adj	lower	upper	reject
Combination	Cycling	0.058	0.002	0.019	0.097	True
Combination	Mixing	0.071	0.000	0.032	0.110	True
Combination	Mono A	0.009	0.980	-0.031	0.048	False
Combination	Mono B	0.069	0.000	0.030	0.108	True
Combination	No treatment	0.206	0.000	0.167	0.245	True
Cycling	Mixing	0.013	0.884	-0.026	0.052	False
Cycling	Mono A	-0.049	0.009	-0.088	-0.010	True
Cycling	Mono B	0.011	0.937	-0.028	0.051	False
Cycling	No treatment	0.148	0.000	0.109	0.188	True
Mixing	Mono A	-0.062	0.001	-0.102	-0.023	True
Mixing	Mono B	-0.002	1.000	-0.041	0.037	False
Mixing	No treatment	0.135	0.000	0.096	0.174	True
Mono A	Mono B	0.060	0.001	0.021	0.100	True
Mono A	No treatment	0.198	0.000	0.158	0.237	True
Mono B	No treatment	0.137	0.000	0.098	0.176	True

Table S51. ***Maximum-emergence*** **scenario: Effect of treatment strategies on the frequency of newly emerging double resistance (ANOVA).**

	Sum of Squares	df	Mean Square	F	Sig.
Between Groups	0.035	5	0.007	41.272	< 0.001
Within Groups	0.010	60	< 0.001		
Total	0.045	65			

Table S52. ***Maximum-emergence*** **scenario: Multiple comparison between the effects of treatment strategies on the frequencies of newly emerging double resistance using Tukey's post-hoc analysis.**

group1	group2	meandiff	p-adj	lower	upper	reject
Combination	Cycling	0.013	0.227	-0.004	0.029	False
Combination	Mixing	0.021	0.004	0.005	0.038	True
Combination	Mono A	0.003	0.990	-0.013	0.020	False
Combination	Mono B	0.024	0.001	0.008	0.041	True
Combination	No treatment	0.070	0.000	0.053	0.086	True
Cycling	Mixing	0.009	0.597	-0.007	0.025	False
Cycling	Mono A	-0.009	0.569	-0.026	0.007	False
Cycling	Mono B	0.012	0.308	-0.005	0.028	False
Cycling	No treatment	0.057	0.000	0.041	0.073	True
Mixing	Mono A	-0.018	0.022	-0.035	-0.002	True
Mixing	Mono B	0.003	0.997	-0.014	0.019	False
Mixing	No treatment	0.048	0.000	0.032	0.065	True
Mono A	Mono B	0.021	0.005	0.004	0.037	True
Mono A	No treatment	0.066	0.000	0.050	0.083	True
Mono B	No treatment	0.045	0.000	0.029	0.062	True

Table S53. ***Maximum-emergence*** **scenario: Effect of treatment strategies on the frequency of superinfections (ANOVA).**

	Sum of Squares	df	Mean Square	F	Sig.
Between Groups	0.019	5	0.004	11.731	< 0.001
Within Groups	0.017	52	< 0.001		
Total	0.036	57			

Table S54. ***Maximum-emergence*** **scenario: Multiple comparison between the effects of treatment strategies on the frequencies of superinfections using Tukey's post-hoc analysis.**

group1	group2	meandiff	p-adj	lower	upper	reject
Combination	Cycling	0.024	0.202	-0.007	0.055	False
Combination	Mixing	0.036	0.015	0.005	0.067	True
Combination	Mono A	0.035	0.021	0.004	0.066	True
Combination	Mono B	0.026	0.160	-0.005	0.057	False
Combination	No treatment	0.068	0.000	0.037	0.099	True
Cycling	Mixing	0.011	0.674	-0.011	0.034	False
Cycling	Mono A	0.010	0.768	-0.013	0.033	False
Cycling	Mono B	0.002	1.000	-0.022	0.025	False
Cycling	No treatment	0.043	0.000	0.021	0.066	True
Mixing	Mono A	-0.001	1.000	-0.024	0.021	False
Mixing	Mono B	-0.010	0.810	-0.033	0.013	False
Mixing	No treatment	0.032	0.001	0.009	0.055	True
Mono A	Mono B	-0.009	0.881	-0.032	0.015	False
Mono A	No treatment	0.033	0.001	0.011	0.056	True
Mono B	No treatment	0.042	0.000	0.019	0.065	True

Table S55. ***Maximum-emergence*** **scenario: Effect of drug ϑ on the frequency of emergence per superinfection (ANOVA).**

	Sum of Squares	df	Mean Square	F	Sig.
Between Groups	10.194	3	3.398	143.661	< 0.001
Within Groups	1.443	61	0.024		
Total	11.637	64			

Table S56. ***Maximum-emergence*** **scenario: Multiple comparison between the effects of drug ϑ on the frequencies of emergence per superinfection using Tukey's post-hoc analysis.**

group1	group2	meandiff	p-adj	lower	upper	reject
A	AB	-0.082	0.752	-0.300	0.136	False
A	B	0.740	0.000	0.624	0.855	True
A	none	0.857	0.000	0.711	1.002	True
AB	B	0.822	0.000	0.602	1.042	True
AB	none	0.939	0.000	0.701	1.176	True
B	none	0.117	0.174	-0.032	0.266	False

References

1. S Tschudin-Sutter, et al., Prospective validation of cessation of contact precautions for extended-spectrum β-lactamase-producing Escherichia coli1. *Emerg. Infect. Dis.* **22**, 1094–1097 (2016).
2. JS Huisman, et al., The effect of sequencing and assembly on the inference of horizontal gene transfer on chromosomal and plasmid phylogenies. *Philos. Trans. R. Soc. B Biol. Sci.* **377** (2022).
3. T Fehér, et al., Competition between transposable elements and mutator genes in bacteria. *Mol. Biol. Evol.* **29**, 3153–3159 (2012).
4. A Carattoli, et al., Identification of plasmids by PCR-based replicon typing. *J. Microbiol. Methods* **63**, 219–228 (2005).
5. JS Huisman, et al., Estimating plasmid conjugation rates: A new computational tool and a critical comparison of methods. *Plasmid* **121**, 102627 (2022).
6. P Virtanen, et al., SciPy 1.0: fundamental algorithms for scientific computing in Python. *Nat. Methods* **17**, 261–272 (2020).
7. S Seabold, J Perktold, Statsmodels: Econometric and Statistical Modeling with Python in *Proc. 9th Python Sci. Conf.* pp. 92–96 (2010).
8. JE Cates, R Christie, LP Garrod, Penicillin-resistant subacute bacterial endocarditis treated by a combination of penicillin and streptomycin. *Br. Med. J.* **31**, 1120–1120 (1951).
9. E Jawetz, JB Gunisson, VR Coleman, Observations on the mode of action of Synergism and Antagonism. *Antibiot. Annu.* **5**, 520–528 (1954).
10. PS Ocampo, et al., Antagonism between bacteriostatic and bactericidal antibiotics is prevalent. *Antimicrob. Agents Chemother.* **58**, 4573–4582 (2014).
11. SA Angermayr, et al., Growth-mediated negative feedback shapes quantitative antibiotic response. *Mol. Syst. Biol.* **18**, 1–19 (2022).
